# Misannotation Awareness: A Tale of Two Gene-Groups

**DOI:** 10.3389/fpls.2016.00868

**Published:** 2016-06-16

**Authors:** Tania Nobre, M. Doroteia Campos, Eva Lucic-Mercy, Birgit Arnholdt-Schmitt

**Affiliations:** ^1^EU Marie Curie Chair, Instituto de Ciências Agrárias e Ambientais Mediterrânicas, Universidade de ÉvoraÉvora, Portugal; ^2^INOQ GmbHSchnega, Germany

**Keywords:** gene family, databases, gene annotation, signature-based classification, phylogeny, alternative oxidase, plastoquinol terminal oxidase

## Abstract

Incorrectly or simply not annotated data is largely increasing in most public databases, undoubtedly caused by the rise in sequence data and the more recent boom of genomic projects. Molecular biologists and bioinformaticists should join efforts to tackle this issue. Practical challenges have been experienced when studying the alternative oxidase (AOX) gene family, and hence the motivation for the present work. Commonly used databases were screened for their capacity to distinguish AOX from the plastid terminal oxidase (also called plastoquinol terminal oxidase; PTOX) and we put forward a simple approach, based on amino acids signatures, that unequivocally distinguishes these gene families. Further, available sequence data on the AOX family in plants was carefully revised to: (1) confirm the classification as AOX and (2) identify to which AOX family member they belong to. We bring forward the urgent need of misannotation awareness and re-annotation of public AOX sequences by highlighting different types of misclassifications and the large under-estimation of data availability.

## General background

It has become clear that the coding portions of the genome are organized hierarchically in gene families and superfamilies. Groups of genes encoding proteins showing similarity with each other, traditionally defined by >50% pairwise amino acid similarity (Thornton and Desalle, 2000), are referred to as a gene family, and arise from general duplication or by duplication and shuffling of exons from different genes (partial gene duplication) followed by divergence. In the same way as the nested organization of living organisms, gene families can show great diversity: many families have just a few very similar genes, others encompass a large number of closely related and distant genes and still others have hundreds of almost identical copies. This diversity in family structure reflects their evolutionary history, their function and their regulation. Thorough classification of genes into gene families allows all types of inference about the evolution of genes and their encoded proteins (Thornton and Desalle, 2000; Demuth et al., 2006). The first classification efforts of proteins have been curated experimentally, but the increase in sequence data and the recent boom of genomic data makes it impossible to keep the same level of accuracy in gene family classification (e.g., Frech and Chen, 2010; Riesenfeld and Pollard, 2013). This is a widely recognized challenge, and bioinformaticists have increased research efforts to provide algorithms for accurate automatic classifications (e.g., Moriya et al., 2007; Schnoes et al., 2009; Pedruzzi et al., 2013; Fox et al., 2014; Szklarczyk et al., 2015) and high-throughput functional analysis of large sets of protein sequences (e.g., Roy et al., 2012; Cozzetto et al., 2013; Szklarczyk et al., 2015). However, in parallel with this effort, there is a need for greater awareness of the specialists that produce and use the data and of those who can actually change the mis-annotations. Genome annotation is an intrinsically complex process, with many different pipelines that focus mainly on the annotation of protein-coding genes; but all pipelines comprise a homology search step (see Yandell and Ence, 2012). Regardless of how the sequences are obtained (traditional Sanger sequencing or high-throughput) gene identification is mainly inferred by homology, which means that the outcome of the inference is never better than the reference(s) itself. This downstream impact goes beyond the annotation as it affects evolutionary studies and the biological understanding of an organism, as well as analyses of pathways, systems, and metabolic processes (Klimke et al., 2011).

This article intends to bring into light the issue of the incorrectly annotated data through a practical -and by the authors experienced- approach when studying a specific gene family in a taxonomical restrict group, the case study of AOX in plants. It targets primarily molecular plant researchers and other specialists that either work directly with these two gene-groups or are facing similar challenges. They are the ones having the deep knowledge on the gene families that they work with and hence are able to provide the data that bioinformaticists need.

## The AOX family in plants–a case study

Alternative oxidase (AOX; EC 1.10.3.11 ubiquinol:O2 oxidoreductase id IPR002680) is a terminal quinol oxidase found in the mitochondria of a wide variety of species from different kingdoms (McDonald, 2008). In plants, it is often present as a small multigene family; e.g., AOX in *Arabidopsis* is encoded by a multigene family with five members: AOX1a, AOX1b, AOX1c, AOX1d, and AOX2 (Saisho et al., 1997, 2001; Clifton et al., 2006).

Across kingdoms, there is a lack of a general pattern with respect to intron/exon structure in AOX genes (McDonald et al., 2015), and the current view is that AOX arose in prokaryotes and entered the eukaryotic lineage via the primary endosymbiotic event that led to the origin of mitochondria (Finnegan et al., 2003; Atteia et al., 2004; McDonald and Vanlerberghe, 2006). Neimanis et al. (2013) suggest that the evolution to a multigene family might have arisen in plants by a duplication event of a single AOX gene after the separation of the Tracheophyta (vascular plants) from the rest of the Embryophyta. More data is needed to test this hypothesis, which does not exclude the possibility of other gene duplication event(s) or even horizontal gene transfer events, to give rise to the pattern of multigene families as we know it nowadays. Within plants, the most common gene structure for AOX comprises four exons interrupted by three introns, with size conservation for all coding regions except the first exon (Considine et al., 2002; Campos et al., 2009). In higher plants, AOX is encoded by three to five genes distributed in two discrete gene subfamilies termed AOX1 and AOX2 (Whelan et al., 1996; Saisho et al., 1997; Vanlerberghe, 2013). While AOX1 expression is induced by stress stimuli in many tissues and is present in both monocot and eudicot plant species, AOX2 is referred to as being constitutively or developmentally expressed in eudicot species (absent from monocots, probably due to gene loss). Even though this paradigm begins to be challenged (Clifton et al., 2006; Costa et al., 2010; Cavalcanti et al., 2013), the current view is still that the subfamilies have different physiological roles. They are thus expected to have evolved under different selection pressures. Costa et al. (2014) have produced the most recent classification scheme of AOX in Angiosperms, and they show that there is an urgent need to reannotate previously named AOXs. This effort needs to be expanded for plants in general and, ideally, for AOX in all kingdoms. With the present work, we call for awareness on careful annotation of public AOX sequences by highlighting misclassifications at different levels.

### AOX vs. PTOX, both membrane-bound di-iron carboxylate proteins

AOX, a non-proton motive ubiquinol oxidase, belongs to the di-iron carboxylate protein superfamily which includes members that are soluble in the cytosol and members that are membrane-bound. AOX and plastid terminal oxidase (PTOX; EC 1.10.3.11 ubiquinol:O2 oxidoreductase id IPR002680) are the membrane-bound members of this superfamily. Whereas AOX is present in almost all phyla, PTOX appears limited to organisms capable of oxygenic photosynthesis (McDonald and Vanlerberghe, 2006). Both are quinol oxidases, but the first is located in the mitochondrial inner membrane at the mitochondria, is involved in the respiratory electron transport and interacts with ubiquinol; the second locates in the stroma, is active in the photosynthetic electron transport and it catalyzes the oxidation with plastoquinol (on AOX role see Vanlerberghe, 2013; on PTOX role see Nawrocki et al., 2015; Krieger-Liszkay and Feilke, 2016). Often, these two family members are mis-annotated in sequence databases.

In a quick survey on sequences deposited at NCBI (using a known AOX sequence and the BLAST tool) at least seven protein sequences, from either gene-discovery sequencing projects or gene re-sequencing, and six sequences originating from whole genome analysis, are annotated as AOX but they are indeed PTOX (Supplementary Data 1). Still other sequences are annotated as immutans, often used as a synonym for PTOX (probably because the immutans variegation mutant of *Arabidopsis* does not have a fully functional PTOX; Wu et al., 1999). Yet, another nine entries that correspond to NCBI annotations appear as alternative oxidase 4 or ubiquinol oxidase 4. Not all this annotations are actually wrong (for instances, the use of immutans to refer to PTOX), but the non-conformity of the nomenclature only raises confusion and often is the cause of the propagation of error. See for example the accession ABD32645 from 2006, a protein sequence from *Medicago truncatula* annotated as encoding for the product alternative oxidase (although it belongs to PTOX, see reasons below). This was spread to more recent data originated from *Medicago* genome sequencing (see accession AES64415) because both traditional sequencing, and certainly genome sequencing projects, use as reference previously annotated sequences.

How to proceed then? There are several compelling reasons to use deduced protein sequences, rather than nucleotide sequences, to determine gene identification for annotation purposes (including the availability of good and maintained databases of protein sequences: e.g., Mulder and Apweiler, 2002; Hunter et al., 2009; Sigrist et al., 2013; Finn et al., 2014). Homology searches based on protein sequences lowers the signal-to-noise ratio in sequence searches and alignments, and eases the distinction of true homology from random similarity because of the close relation between protein sequence and function. Protein sequences can be analyzed fully, but to rapidly define the family of proteins to which the sequence belongs to it is practical to look for single domains or motifs. These motifs have been shown to be important for protein functionality and can outline a family of proteins (Martinez, 2013), being thus useful for implementation in protein classification systems. The large influx of raw sequence data from genome sequencing projects has led to the emergence of numerous automatic methods for protein sequence analysis and classification, based on comparative analyses. These bioinformatic tools largely rely on the identification of motifs that could be previously encountered in characterized protein families. Two main types of databases are available, (1) the traditional gene family databases trusting on signatures such as Pfam (Finn et al., 2014) or PROSITE (Sigrist et al., 2013) and (2) the integrative databases such as InterPro (Hunter et al., 2009) and CDD (Marchler-Bauer et al., 2011) joining information from several major signature databases (Martinez, 2011, 2013). Signature-based methods are routinely used for gene function annotation but they seem to have a limitation in the case of distinguishing between AOX/PTOX: whereas all databases return sequences with high homologies to the sequence of interest, their classification is not consensual and most fail to clearly distinguish these two sub-families (Supplementary Table 1).

**Figure 1:**
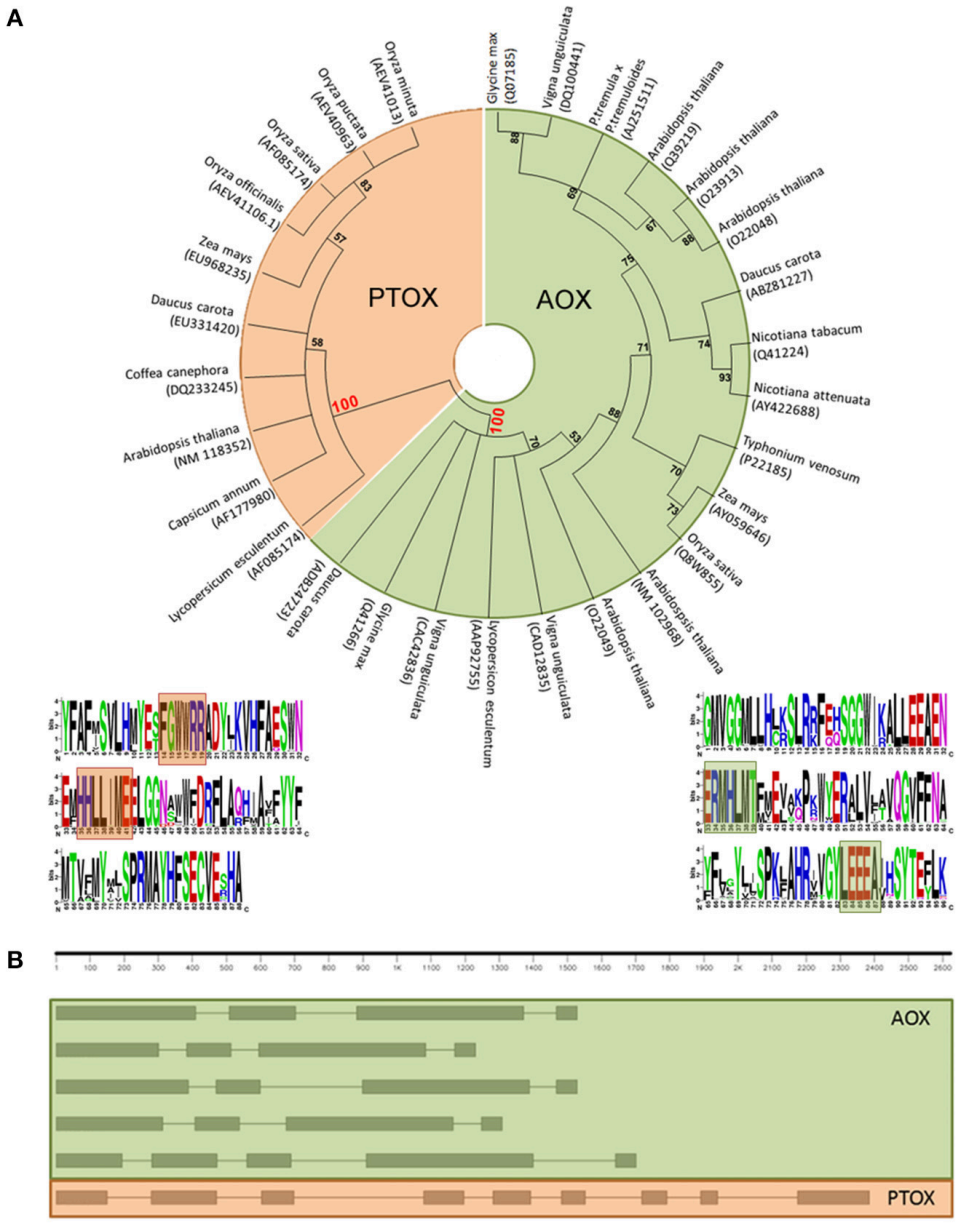
**(A)** Neighbor-joining (NJ) tree of PTOX sequences (orange) and AOX sequences (green), clearly showing that they belong to two separate clades. Logo representation of the AOX/PTOX signature was constructed at the web interface program weblogo (Crooks et al., 2004). We propose two fingerprints group specific: (1) for PTOX, based on 60 sequences, (F)GWWRR and HHLL(I)ME; (2) for AOX, based on 206 sequences, ERMHLVT and YLEEEA (Supplementary Data 2). **(B)** Gene structure of AOX and PTOX *Arabidopsis thaliana* nucleotide sequence. AOX in plants generally presents 4 exons interrupted by 3 introns (evolutionary intron loss or gain resulted in the variation of intron numbers in some AOX members, Cardoso et al., 2015): in order of appearance, AT1G32350; AT3G22360; AT3G22370; AT3G27620; AT5G84210. PTOX is typically structured in 9 exons and 8 introns: AT4G22260.

Actually, a multiple sequence alignment of full-length amino acid sequences of the putative gene models, as well as characterized sequences, followed by a neighbor-joining clustering showed that AOX and PTOX form clearly different clades (Figure 1A). We have found two sites that seem to show a specific conservation of amino acids related to the subfamily members–fingerprints–and we propose their use as a quick and efficient way to distinguish plant AOX from PTOX sequences. These signatures may serve as identification motifs specific for the two subfamilies, and when scanned against the GenBank database, retrieved only members of each subfamily. These sequences can be used to identify additional members of the two subfamilies in other plant species as their genomes are being sequenced. The general structure of both AOX and PTOX is different and on itself might represent an extra way to verify gene affiliation of already published sequences to either AOX or PTOX family member (Figure 1B).

### Members of the AOX family

The naming of AOX genes originally occurred in the order of their discovery in a species but the need of a classification system became obvious as more sequences were made available. Considine et al. (2002) provided an initial classification that divided plant AOX in two subfamilies (AOX1 and AOX2) while in non-plant species the AOX was named as AOX0. Recently, Costa et al. (2014) proposed a classification scheme for AOX in angiosperms based on protein tree topologies, the analysis of specific amino acid sites found to differ between AOX subfamilies and subtypes, and the known evolutionary history of angiosperms.

**Figure 2:**
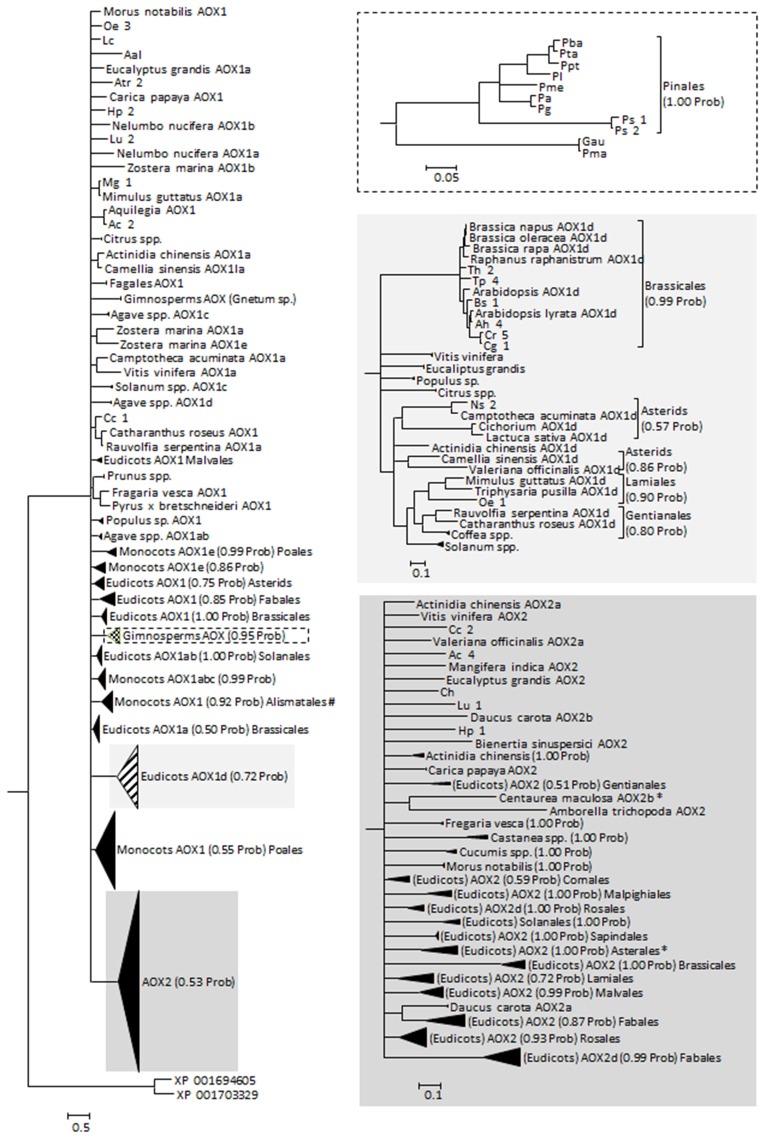
**Reconstructed phylogeny of plants at the gene family AOX included in this study (a combination of newly collected sequences and the alignment made available by Costa et al., 2014)**. The optimal substitution model was selected in MrModeltest 2.2 (Posada and Crandall, 1998) as being the JTT+I+G. The phylogeny corresponds to the majority rule consensus tree of trees sampled in a Bayesian analysis (conducted using MrBayes version 3.0 (Huelsenbeck and Ronquist, 2001; Ronquist and Huelsenbeck, 2003); with default settings and with MCMC—considering 100, 000 generations—runs being repeated three times as a safeguard against spurious results; first 1000 trees were discarded as burn-in; stationarity was confirmed by analysis of the log-likelihoods and the consistency between runs). The numbers above the branches refer to the Bayesian posterior probability of the nodes (more than 50%) derived from 19500 Markov chain Monte Carlo-sampled trees. #Clade containing a putative *Solanum tuberosum* AOX1 sequence (sequence id PRF: 1588565); likely a misidentification of the organism. *Centaurea maculosa (Asterales) AOX2b sequence (GenBank EH723572.1) does not cluster with the other Asterales.

The protein alignments made available by Costa et al. (2014) as Supplementary Material were used as the base data for our phylogenetic analysis. To the dataset, other sequences retrieved from the different databases were added including the few existing data on Gymnosperms, giving a total of 369 sequences. The sequences retrieved in this work were analyzed for the presence/absence of the specific amino acid motifs and a tentative AOX classification was constructed (Supplementary Table 2). Most of those sequences were obtained from genome sequencing projects and were often not annotated. Adding to the 32 mis-annotated sequences found by Costa et al. (2014), we found 13 more mis-annotated sequences (in 120 sequences, of which 73 were without any annotation and 22 were referred to simply as AOX). Altogether, 369 sequences were used on the phylogenetic analysis (Figure 2; see Supplementary Data 3) and their classification was adjusted. The phylogeny returned is largely unresolved, suggesting an almost simultaneous divergence from a common ancestor, or simply a lack of information to resolve the polytomies. These polytomies can also be the result of convergent evolution and/or of recent gene duplication (both previously suggested for AOX, e.g., Neimanis et al., 2013; Costa et al., 2014). Classification and annotation can be further hindered by the observation that some plants that are more phylogenetically divergent have similar multigene families representatives (e.g., *Arabidopsis* and poplar, a Brassicales and a Malpighiales, both have AOX1a, AOX1b, AOX1c, and AOX1d), while more closely related species have large differences in AOX family composition (e.g., *Arabidopsis* and papaya, both Brassicales, the first with all known members of AOX1 and the last with just one AOX1 gene; Costa et al., 2014; Cardoso et al., 2015).

### Short note on gene isolation and annotation

When starting the isolation process of a specific gene in a species where no further information is available, the most common laboratory strategy has been the use of “universal primers” (when existing). In the case of AOX, the most commonly used primers are the ones described by Saisho et al. (1997). These seem to work well across kingdoms and, given what we know about the evolutionary story of AOXs, also across family members. After database searches for DNA sequences encoding for the different AOX genes and an *in silico* amplification of the region comprised between the primers P1 and P2 as probes (Saisho et al., 1997), we have got 218 sequences of the expected 444 bp amplicon (with no insertions or deletions; Supplementary Data 4). The resulting NJ tree highlights that, with this conserved fragment, it is possible to discriminate between AOX1 and AOX2 but no other gene member can be clearly identified (Supplementary Figure 1) and should thus not be further annotated only on the basis of fragment homology.

## Final considerations

Focusing on the membrane-bound di-iron carboxylate proteins, we show that a re-annotation is needed. We have reviewed a large quantity of data present in different databases, identified an easy way (based on signatures) of distinguishing between the two mis-classified gene families—AOX vs. PTOX—and presented the largest phylogeny to date that comprises curated and annotated AOX amino acids sequences (based on the system developed by Costa et al., 2014) as well as newly identified sequences from genomic databases. In the particular case of AOX, we realize that the great majority of data available is “hidden” in contigs and scaffoldings as non-identified regions. If at one side this implies that there are fewer non-spotted misannotations than originally thought, it also means that the amount of data available for this gene family is largely under-estimated.

Misannotation in superfamilies containing multiple families that catalyze different reactions is an issue needing to be addressed as it can have serious repercussions on data interpretation and ultimately on our understanding of the systems. It is a shared responsibility, of researchers working with these superfamilies and bioinformaticists to tackle this challenge. The current identification and classification system is prone to error propagation and an increase in annotation errors over time is to be expected. Researcher's awareness can minimize error propagation and certainly errors in data interpretation. It is also a dynamic process that needs to be revised and updated in frame of the continuous new flow of data. The ones with deeper knowledge on the specific genes families can positively contribute to data revision and collaborate with bioinformaticists to the updating of the classification systems.

## Author contributions

TN and BA-S conceptualized the manuscript. TN, MDC, and EL-M collected and analyzed the data. TN designed and wrote the manuscript and all the authors revised and approved it.

### Conflict of interest statement

The authors declare that the research was conducted in the absence of any commercial or financial relationships that could be construed as a potential conflict of interest.
