# Supplementary material for: Misannotation Awareness: A Tale of Two Gene-Groups
Source: Front Plant Sci. 2016 Jun 16;7:868. doi: 10.3389/fpls.2016.00868 (PMC4909761; doi:10.3389/fpls.2016.00868)
Supplement: Supplementary file 1 [file Table1.DOCX]

**Table 1:** Signature databases and their capacity to distinguish between AOX and PTOX according to similarities with known sequence signatures.

| **Tool** | **Entries found *** | **Remarks** | **Ref.** |
| --- | --- | --- | --- |
| [CDD](http://www.ncbi.nlm.nih.gov/Structure/cdd/cdd.shtml) | Cd01053 | No distinction AOX-PTOX | (Marchler-Bauer et al., 2011) |
| [eggNOG](http://eggnog.embl.de/version_4.0.beta/index.html) | Several groups/fingerprints | Recognition of a different motif for PTOX, but annotated as AOX only | (Powell et al., 2014) |
| [InterPro](http://www.ebi.ac.uk/interpro/) | IPR002680 | No distinction AOX-PTOX | (Hunter et al., 2009) |
| [OMA](http://omabrowser.org/oma/home/) | Several groups/fingerprints | Recognition of specific PTOX fingerprints but not accurate  (most PTOX are annotated as AOX) | (Altenhoff et al., 2011) |
| [PANTHER](http://www.pantherdb.org/) | PTHR31803 : Ubiquinol oxidases  AOX: SF3, SF4, SF5, SF6, SF7, SF8 and SF9  PTOX: SF2, SF10 | Recognition of a different motif for PTOX and AOX but unclear subfamily classification within PTHR31803 family | (Mi et al., 2013) |
| [Pfam](http://pfam.xfam.org/) | Pfam01786 | No distinction AOX-PTOX | (Finn et al., 2014) |
| [Prodom](http://prodom.prabi.fr/prodom/current/html/home.php) | Several families | Recognition of a different motif for PTOX, but annotated as AOX only | (Servant et al., 2002) |
| [Prosite](http://prosite.expasy.org/prosite.html) | - | No AOX-PTOX sequences referenced | (Sigrist et al., 2013) |
| [Superfamily](http://supfam.cs.bris.ac.uk/SUPERFAMILY/index.html) | - | No AOX-PTOX sequences referenced | (Gough, 2002) |
| [UniProtKB/ UniRef](http://www.uniprot.org/) |  | Search by keywords: no distinction AOX-PTOX | (Consortium, 2014) |

* *Blast, Advanced Search and Gene Ontology tools were used at several genomes, protein and integrative databases to identify gene models. Additional searches were performed to probe genome databases with the use of a range of sequences of available in GenBank (http://www.ncbi.nlm.nih.gov). All detected gene models were inspected manually. The retrieved sequences were used for database searches by blast, and for phylogenetical analyses.*

Altenhoff, A. M., Schneider, A., Gonnet, G. H., and Dessimoz, C. (2011). OMA 2011: orthology inference among 1000 complete genomes. *Nucleic Acids Res.* 39, D289–94. doi:10.1093/nar/gkq1238.

Consortium, T. U. (2014). UniProt: a hub for protein information. *Nucleic Acids Res.*, 1–9. doi:10.1093/nar/gku989.

Finn, R. D., Bateman, A., Clements, J., Coggill, P., Eberhardt, R. Y., Eddy, S. R., et al. (2014). Pfam: the protein families database. *Nucleic Acids Res.* 42, D222–30. doi:10.1093/nar/gkt1223.

Gough, J. (2002). The SUPERFAMILY database in structural genomics. *Acta Crystallogr. Sect. D Biol. Crystallogr.* 58, 1897–1900. doi:10.1107/S0907444902015160.

Hunter, S., Apweiler, R., Attwood, T. K., Bairoch, A., Bateman, A., Binns, D., et al. (2009). InterPro: the integrative protein signature database. *Nucleic Acids Res.* 37, D211–5. doi:10.1093/nar/gkn785.

Marchler-Bauer, A., Lu, S., Anderson, J. B., Chitsaz, F., Derbyshire, M. K., DeWeese-Scott, C., et al. (2011). CDD: a Conserved Domain Database for the functional annotation of proteins. *Nucleic Acids Res.* 39, D225–9. doi:10.1093/nar/gkq1189.

Mi, H., Muruganujan, A., and Thomas, P. D. (2013). PANTHER in 2013: modeling the evolution of gene function, and other gene attributes, in the context of phylogenetic trees. *Nucleic Acids Res.* 41, D377–86. doi:10.1093/nar/gks1118.

Powell, S., Forslund, K., Szklarczyk, D., Trachana, K., Roth, A., Huerta-Cepas, J., et al. (2014). eggNOG v4.0: nested orthology inference across 3686 organisms. *Nucleic Acids Res.* 42, D231–9. doi:10.1093/nar/gkt1253.

Servant, F., Bru, C., Peyruc, D., and Kahn, D. (2002). ProDom : Automated clustering of homologous domains. 3, 246–251.

Sigrist, C. J. a, de Castro, E., Cerutti, L., Cuche, B. a, Hulo, N., Bridge, A., et al. (2013). New and continuing developments at PROSITE. *Nucleic Acids Res.* 41, D344–7. doi:10.1093/nar/gks1067.
