# Supplementary material for: Misannotation Awareness: A Tale of Two Gene-Groups
Source: Front Plant Sci. 2016 Jun 16;7:868. doi: 10.3389/fpls.2016.00868 (PMC4909761; doi:10.3389/fpls.2016.00868)
Supplement: Supplementary file 3 [file DataSheet1.DOCX]

**From traditional sequencing projects**

>AAG00450.1

tppeeeggfdeeltlagedgdwvvrfeqsfnvfltdtvifildilyrdrdyarffvletiarvpyfafisvlhlyetfgwsrradnikvhfaesmnefhhllimealggnsvwldrflarfsaffyyfvtvgmymlsprmayhfsecverhaystydkflklngeelkklpapevavnyymnedlymfdefqtsrapnsrrpkvdnlydvfvnvrddersiarq

>EOY28837.1

mdpktcqtkahddincckqraatrrcahpnrlslknyqttgrsrlpltfvhrslplcslgfevsyflkhagemttaslssaafatsvsssfrarnsrtslpsnyqnplrcssppsyrplsgklypvkatllqedeeevvveksfrtkgfpgneveegwesranssssdlerwvikveqsvnvfltdsvikildtlyhdrdyprffvletiarvpyfafisvlhmyesfgwwrradylkvhfaeswnemhhllimeelggnswwfdrflaqhiaifyyimtvfmyaisprmayhfsecveshafetydkfirakgkelkekpapevaikyytggdlylfdefqterapcsrrpkienlydvfvnirddeaehcktmkacqthgnlrsphsypvdgfedmpgcmipeancegivdcikksltpsqvkqkeei

>EOY28838.1

mttaslssaafatsvsssfrarnsrtslpsnyqnplrcssppsyrplsgklypvkatllqedeeevvveksfrtkgfpgneveegwesranssssdlerwvikveqsvnvfltdsvikildtlyhdrdyprffvletiarvpyfafisvlhmyesfgwwrradylkvhfaeswnemhhllimeelggnswwfdrflaqhiaifyyimtvfmyaisprmayhfsecveshafetydkfirakgkelkekpapevaikyytggdlylfdefqterapcsrrpkienlydvfvnirddeaehcktmkacqthgnlrsphsypvdgfedmpgcmipeancevllegpkqsfqwsnwnlasd

>ABD32645.1 mastamfssslfpitplnklsssrnslifrplsfrpplfrirssllqdkedkvitqntfpsktspldsvtenstndddtsstsawekgvikveqsvnifltdsvikildalyrdrnyarffvletiarvpyfafmsilhmyesfgwwrradylkvhfaeswnemhhllimeelggnawwfdrflaqhiaifyyfmtalmylisprmayhfsecveshafetydkfikeqgeelkkmpapevavnyytggdlylfdefqtsrvpntrrptidnlydvflnirddeaehcktmracqtygnlrsphsyadaedddesvctieagcegivdcikksvtsnpakvk

>EEF43798.1

matmsisptttaavyaisnskalssfssrnglsfislssprfkatgklcrvqatvlreneekvvidetfqpksftddddgkggrtggdppdtslerwvikleqsvnvfltdsvikildafyhdrdyarffvletiarvpyfafmsvlhmyesfgwwrradylkvhfaeswnemhhllimeelggnswwfdrflaqhiaiiyyimtvfmyalsprmayhfsecveshayatydkfikaqgeelkklpapevavkyytegdlylfdefqtsraphsrrpkidnlydvflnirddeaehcktmracqthgnlrsphshvendiedvsgcilpeadcegivdcmkksltsapskeeirgg

>XP_002518411.1

matmsisptttaavyaisnskalssfssrnglsfislssprfkatgklcrvqatvlreneekvvidetfqpksftddddgkggrtggdppdtslerwvikleqsvnvfltdsvikildafyhdrdyarffvletiarvpyfafmsvlhmyesfgwwrradylkvhfaeswnemhhllimeelggnswwfdrflaqhiaiiyyimtvfmyalsprmayhfsecveshayatydkfikaqgeelkklpapevavkyytegdlylfdefqtsraphsrrpkidnlydvflnirddeaehcktmracqthgnlrsphshvendiedvsgcilpeadcegivdcmkksltsapskeeirgg

>Q56X52.2

maaisgissgtltisrplvtlrrsraavsyssshrllhhlplssrrlllrnnhrvqatilqddeekvvveesfkaetstgtepleepnmsssstsafetwiikleqgvnvfltdsvikildtlyrdrtyarffvletiarvpyfafmsvlhmyetfgwwrradylkvhfaeswnemhhllimeelggnswwfdrflaqhiatfyyfmtvflyilsprmayhfsecveshayetydkflkasgeelknmpapdiavkyytggdlylfdefqtsrtpntrrpvienlydvfvnirddeaehcktmracqtlgslrsphsiledddteeesgcvvpeeahcegivdclkksits

**From whole genome sequencing projects**

>DAA35460.1

mavastsplsakpatapsppapgsgllalgvrrapataawrrlrveairtqrtevpveesapardaaaaapldgngagadgsvvpssddswvvkleqsfnifatdsvimvlkgvygdryyarffaletiarvpyfafisvlhlyatfgwwrradyikvhfaqswnefhhllimeelggdslwfdcflarfmaffyyfmtvamymlsprmayhfsecverhaystydeflklheeelkrlpapeaalnyymnedlylfdefqasrtpgsrrpkidnlydvfvniredeaehcktmktcqthgnlrsphstpncleddtecvipendcegivdcvkksltk

>DAA35461.1

mavastsplsatapsppapvsgflalparrgcatrlgsaaawrrlrveaiwkqqekqraevsveepapvreaaapldgvgaddpmvpssdeswvvrleqsvnifltesviillntvyrdrnyarffvletiarvpyfafisvlhmyetfgwwrradylkvhfaqslnefhhllimeelggnaiwidcflarfmaffyyfmtvamymlsprmayhfsecverhaystydkflklheeelktlpapeaalnyylnedlylfdefqttripcsrrpkidnlydvfvnirddeaehcktmkacqthgtlrsphsmpncleaatecvipendcegivdcvkksltk

>NP_567658.1 maaisgissgtltisrplvtlrrsraavsyssshrllhhlplssrrlllrnnhrvqatilqddeekvvveesfkaetstgtepleepnmsssstsafetwiikleqgvnvfltdsvikildtlyrdrtyarffvletiarvpyfafmsvlhmyetfgwwrradylkvhfaeswnemhhllimeelggnswwfdrflaqhiatfyyfmtvflyilsprmayhfsecveshayetydkflkasgeelknmpapdiavkyytggdlylfdefqtsrtpntrrpvienlydvfvnirddeaehcktmracqtlgslrsphsiledddteeesgcvvpeeahcegivdclkksits

>EMT19344.1

maaralagagravlslpsvrrratnswaavrdtffstkevfeshrvvftvgtsiasvltawagyslrhmqqtkidkrlhsieeslrnthkvehdeikkivtsynistsaciataltttvvgyalgwrggawytrrivrreqqklmgqiksqnrrgrriraeatartrqekeqqeaevsavedsfavreaaaapppeeeggfdeeltlagedgdwvvrfeqsfnvfltdtvifildilyrdrdyarffvletiarvpyfafisvlhlyetfgwsrradnikvhfaesmnefhhllimealggnsvwldrflarfsaffyyfvtvgmymlsprmayhfsecverhaystydkflklngeelkklpapevavnyymnedlymfdnlydvfvnvrddeaehcktmkacqthetlrsphavqssieadae

>EPS61569.1

mesaltvsgsaalpvwaaafppqlsselgflkfnqsclqkrrgkkflcirrasgwinqfgnacsnrrkllfqvqattlqeddeevvveksfdpksfpekaegrtddaedesnsfekwiikieqstnifltdsvikildtlyqdrdyarfyvletiarvpyfafmsvlhmyesfgwwrradylkvhfaeswnemhhllimegvdtdvksssvscselggnswwfdrylaqiiailyyfmtvfmytlsprmayhfsecvenhafetydkfvktekeklerlpapavavkyytedlylfdefqtsrppnsrrpkienlydvflnvrddeaehcktmracqthgslrsphlyeddgydsgcaldcegvvdcikksmtdpkl

>AES64415.1

mastamfssslfpitplnklsssrnslifrplsfrpplfrirssllqdkedkvitqntfpsktspldsvtenstndddtsstsawekgvikveqsvnifltdsvikildalyrdrnyarffvletiarvpyfafmsilhmyesfgwwrradylkvhfaeswnemhhllimeelggnawwfdrflaqhiaifyyfmtalmylisprmayhfsecveshafetydkfikeqgeelkkmpapevavnyytggdlylfdefqtsrvpntrrptidnlydvflnirddeaehcktmracqtygnlrsphsyadaedddesvctieagcegivdcikksvtsnpakvk

>XP_003594164.1 mastamfssslfpitplnklsssrnslifrplsfrpplfrirssllqdkedkvitqntfpsktspldsvtenstndddtsstsawekgvikveqsvnifltdsvikildalyrdrnyarffvletiarvpyfafmsilhmyesfgwwrradylkvhfaeswnemhhllimeelggnawwfdrflaqhiaifyyfmtalmylisprmayhfsecveshafetydkfikeqgeelkkmpapevavnyytggdlylfdefqtsrvpntrrptidnlydvflnirddeaehcktmracqtygnlrsphsyadaedddesvctieagcegivdcikksvtsnpakvk
