# Supplementary material for: Misannotation Awareness: A Tale of Two Gene-Groups
Source: Front Plant Sci. 2016 Jun 16;7:868. doi: 10.3389/fpls.2016.00868 (PMC4909761; doi:10.3389/fpls.2016.00868)
Supplement: Supplementary file 4 [file DataSheet2.DOCX]

**PTOX**

>Z. mays IMMUTANS (NM_001157308.1)

YFAFISVLHLYATFGWWRRADYIKVHFAQSWNEFHHLLIMEELGGDSLWFDCFLARFMAFFYYFMTVAMYMLSPRMAYHFSECVERHA

>Z. mays (GRMZM2G102349_T01)

YFAFISVLHMYETFGWWRRADYLKVHFAQSLNEFHHLLIMEELGGNAIWIDCFLARFMAFFYYFMTVAMYMLSPRMAYHFSECVERHA

>Z. mays (GRMZM2G010555_T02)

YFAFISVLHLYATFGWWRRADYIKVHFAQSWNEFHHLLIMEELGGDSLWFDCFLARFMAFFYYFMTVAMYMLSPRMAYHFSECVERHA

>D. carota PTOX (EU331420.2)

YFAFMSVLHMYESFGWWRRADYLKVHFAESWNEMHHLLIMEELGGNAWWFDRFLSQHIAVFYYFMAAFMYLLSPRMAYHFSECVEHHA

>S. bicolor similar to PTOX(Sb06g032180.1)

YFGFISVLHLYETFGWWRRADYIKVHFAQSWNEFHHLLIMEELGGNALWIDRFLARFMAFFYYFMTVAMYMLSPRMAYHFSECVERHA

>S. bicolor similar to PTOX (Sb06g032190.1)

YFAFISVLHMYSTFGWWRRADYIKVHFAQSWNEFHHLLIMEELGGNSLWIDCFLARFMAFFYYFVTVAMYMLSPRMAYHFSECVERHA

>A. coerulea (Aquca_028_00047.1)

YFAFMSVLHMYESFGWWRRADYLKVHFAESWNEMHHLLIMEELGGNAWWFDRFLAQHIAVFYYFMTAFMYLLSPRMAYHFSECVESHA

>B. rapa (Bra013604)

YFAFMSVLHMYETFGWWRRADYLKVHFAESWNEMHHLLIMEELGGNSWWFDRLLGQIVATFYYFMTVFLYIVSPRMAYHFSECVESHA

>S. lycopersicum (Solyc11g011990.1.1)

YFAFISVLHMYESFGWWRRADYMKVHFAESWNEMHHLLIMEELGGNAWWFDRFLAQHIAIFYYFMTVLMYALSPRMAYHFSECVESHA

>A. thaliana (NM_118352.3)

YFAFMSVLHMYETFGWWRRADYLKVHFAESWNEMHHLLIMEELGGNSWWFDRFLAQHIATFYYFMTVFLYILSPRMAYHFSECVESHA

>S. tuberosum (PGSC0003DMT400034708)

YFAFISVLHMYESFGWWRRADYMKVHFAESWNEMHHLLIMEELGGNAWWFDRFLAQHIAIFYYFMTVLMYALSPRMAYHFSECVESHA

>T. cacao (Thecc1EG030324t1)

YFAFISVLHMYESFGWWRRADYLKVHFAESWNEMHHLLIMEELGGNSWWFDRFLAQHIAIFYYIMTVFMYAISPRMAYHFSECVESHA

>G. max (Glyma15g11950.1) YFAFMSVLHMYESFGWWRRADYLKVHFAESWNEMHHLLIMEELGGNAWWFDRFLAQHIAIFYYIMTVLMYAVSPRMAYHFSECVESHA

> G. max (Glyma09g01130.1)

YFAFMSVLHMYESFGWWRRADYLKVHFAESWNEMHHLLIMEELGGNAWWFDRFLAQHIAIFYYIMTVLMYAVSPRMAYHFSECVESHA

>B. distachyon (Bradi5g25540.1)

YFAFISVLHMYETFGWWRRADYIKVHFAESMNEFHHLLIMEELGGNSELVDRFLARFSAFFYYFMTVAMYMLSPRMAYHFSECVERHA

>R. communis (29842.t000019|29842.m003522)

YFAFMSVLHMYESFGWWRRADYLKVHFAESWNEMHHLLIMEELGGNSWWFDRFLAQHIAIIYYIMTVFMYALSPRMAYHFSECVESHA

>V. vinifera (GSVIVG01006689001)

YFAFMSVLHMYESFGWWRRADYLKVHFAESWNEMHHLLIMEELGGNAWWFDRFLAQHIAIFYYFMTVFMYVLSPRMAYHLSECVESHA

>M. domestica (MDP0000195881)

YFAFMSVLHMYESFGWWRRADYLKVHFAESWNEMHHLLIMEELGGNAWWFDRFLAQHIAVFYYFMTAFMYVISPRMAYHFSECVESHA

>P. trichocarpa (Potri.004G002600.1)

YFAFISVLHLYESFGWWRRSDYIKVHFAESWNEMHHLLIMEELGGNSLWFDRFLAQHMAFFYYIMTVLMYALSPRMAYHFSECVENHA

>P. trichocarpa (Potri.011G021800.1)

YFAFISVLHMYESFGWWRRADYLKVHFAESWNEMHHLLIMEELGGNSWWFDRLLAQVIATSYYFMTVLMYALSPRMAYHFSECVESHA

>P. vulgaris (Phvul.006G144800.1)

YFAFMSVLHMYESFGWWRRADYLKVHFAESWNEMHHLLIMEELGGNAWWFDRFLAQHIAIFYYIMTVLMYAVSPRMAYHFSECVESHA

>F. vesca(gene19737-v1.0-hybrid)

YFAFMSVLHMYESFGWWRRSEYLKVHFAESWNEMHHLLIMEELGGNAWWFDRFLAQHIAVFYYFMTVFMYIISPRMAYHFSECVEGHA

>C. papaya (evm.TU.supercontig_152.62)

YFAFMSVLHMYESFGWWRRADYLKVHFAESWNEMHHLLIMEELGGNAWWFDRFLAQHIAFFYYLMTVFMYAISPRMAYHFSECVESHA

>C. sinensis (orange1.1g018864m.g)

YFAFISVLHMYESFGWWRRADYLKVHFAESWNEMHHLLIMEELGGNAWWFDRFLAQHIAVAYYFVTVFMYVISPRMAYHFSECVESHA

>S. italica (Si022681m.g)

YFAFISVLHLYETFGWSRRADYIKVHFAESWNEFHHLLIMEELGGNALFFDRFLARFMAFFYYFMTVGMYMLSPRMAYHFSECVERHA

>T. halophila (Thhalv10025624m.g)

YFAFMSVLHMYETFGWWRRADYLKVHFAESWNEMHHLLIMEELGGNSWWFDRFLAQHIATFYYFMTVFLYIISPRMAYHFSECVESHA

>C. clementine (Ciclev10015546m.g)

YFAFISVLHMYESFGWWRRADYLKVHFAESWNEMHHLLIMEELGGNAWWFDRFLAQHIAVAYYFVTVFMYVISPRMAYHFSECVESHA

>C. rubella (Carubv10005140m.g)

YFAFMSVLHMYETFGWWRRADYLKVHFAESWNEMHHLLIMEELGGNSWWFDRFLAQHIATFYYFMTVFLYIVSPRMAYHFSECVESHA

>E. grandis (Eucgr.E03960.1)

YFAFMSILHMYESFGWWRRADYLKVHFAESWNEMHHLLIMEELGGNAWWFDRFLAQHIAIFYYLMTVFMYALSPRMAYHFSECVESHA

>G. raimondii (Gorai.009G182200.1)

YFAFISVLHMYESFGWWRRADYLKVHFAESWNEMHHLLIMEELGGNSWWFDRFLAQHIAIFYYFMTVFMYTLSPRMAYHFSECVESHA

>L. usitatissimum (Lus10002872.g)

YFAFISVLHLYESVGWWRRADYIKVHFAESWNEMHHLLIMEELGGNSWWFDRFLAQHVAVFYYFMTVFMYALSPRMAYHFSECVENHA

>P. persica (ppa007820m)

YFAFMSVLHMYESFGWWRRADYLKVHFAESWNEMHHLLIMEELGGNAWWFDRFLAQHIAIFYYFMTAFMYIISPRMAYHFSECVEGHA

>M. truncatula (Medtr2g025140.1)

YFAFMSILHMYESFGWWRRADYLKVHFAESWNEMHHLLIMEELGGNAWWFDRFLAQHIAIFYYFMTALMYLISPRMAYHFSECVESHA

>O. sativa (LOC_Os04g57320.1)

YFAFISVLHMYETFGWWRRADYIKVHFAESWNEFHHLLIMEELGGNSLWVDRFLARFAAFFYYFMTVAMYMVSPRMAYHFSECVERHA

>P. mume PTOX (XM_008226514.1)

YFAFMSVLHMYESFGWWRRADYLKVHFAESWNEMHHLLIMEELGGNAWWFDRFLAQHIAIFYYFMTAFMYIISPRMAYHFSECVEGHA

>A. trichopoda (lcl|evm_27.model.AmTr_v1.0_scaffold00074.38)

YFAFMSVLHMYESFGWWRRADYLKVHFAESWNELHHLLIMEELGGNSMWFDRLLAQHIAIFYYFMTVIMYAVSARMAYHFSECVEKHA

>O. brachyantha ubiquinol oxidase 4 (XM_006652926.1)

YFAFISVLHMYETFGWWRRADYIKVHFAESWNEFHHLLIMEELGGNSLWVDRFLARFAAFFYYFMTVAMYMLSPRMAYHFSECVERHA

>M. esculenta (cassava4.1_010671m.g)

YFAFISVLHMYESFGWWRRADYLKVHFAESWNEMHHLLIMEELGGNAWWFDQFLAQHIAIIYYIMTVFMYALSPRMAYHFSECVESHA

>M. guttatus (mgv1a008454m.g)

YFAFMSVLHMYESFGWWRRADYIKVHFAESWNEMHHLLIMEELGGNAWWFDRFLAQHIAVFYYFMTAFMYALSPRMAYHFSECVEGHA

>P. virgatum (Pavirv00023371m.g)

YFAFISVLHMYETFGWWRRADYIKVHFAESWNEFHHLLIMEELGGNALWIDRFLARFMAFFYYFMTVGMYMLSPRMAYHFSECVERHA

>Pavir.Gb00094.1
YFAFISVLHMYETFGWWRRADYIKVHFAESWNEFHHLLIMEELGGNALWIDRFLARFMAFFYYFMTVGMYMLSPRMAYHFSECVERHA

>P. virgatum (Pavirv00046112m.g)

YFAFISVLHMYETFGWWRRADYIKVHFAESWNEFHHLLIMEELGGNALWIDRFLARFMAFFYYFMTVGMYMLSPRMAYHFSECVERHA

>Pavir.Ga00185.1

YFAFISVLHMYETFGWWRRADYIKVHFAESWNEFHHLLIMEELGGNALWIDRFLARFMAFFYYFMTVGMYMLSPRMAYHFSECVERHA

>gi|659118179|ref|XP_008458984.1| PREDICTED: ubiquinol oxidase 4, chloroplastic/chromoplastic [Cucumis melo]

YFAFLSVLHLYESFGWWRRADYLKVHFAESWNEMHHLLIMEELGGNDWWFDRFLAQHIAVAYYFMTVFMYMISPRMAYHLSECVESHA

>gi|659118183|ref|XP_008458986.1| PREDICTED: ubiquinol oxidase 4, chloroplastic/chromoplastic-like [Cucumis melo]

YFAFVSVLHMYESFGWWRRADYLKVHFAESWNEMHHLLIMEELGGNDWWFDRFLAQHIAVAYYFMTVFMYMISPRMAYHFSECVESHA

>C. sativus (Cucsa.332500.1)

YFAFLSVLHLYESFGWWRRADYLKVHFAESWNEMHHLLIMEELGGNDGWFDRFLAQHIAVAYYFMTVFMYMISPRMAYHLSECVESHA

>C. sativus (Cucsa.332520.1)

YFAFVSVLHMYESFGWWRRADYLKVHFAESWNEMHHLLIMEELGGNDWWFDRFLAQHIAVAYYFMTVFMYMISPRMAYHFSECVESHA

>gi|81296552|gb|ABB70513.1| plastid terminal oxidase [Coffea canephora]

YFAFMSVLHLYESFGWWRRADLSEVHFAESWNEMHHLLIMEELGGNSWWFDRFLAQHIAVFYYFMTVFMYMLSPRMAYHFSECVESHA

>P. patens (Pp1s84_283V6.1 synonym:Phypa_130694)

YFAFVSVLNMYESFGWWRRADYLKVHFAESWNELHHLLTMEALGGDERWVDRFLAQHIAVGYYFMTVVMYLLSPRMAYHFSECVKKHA

>S. moellendorffii (15411121_peptide)

YFAFVSVLHMYESFGWWRRADYLKVHFAESWNELHHLLVMEALGGDERWFDRFLAQHIAVAYYFLTSLMYTISPRMAYHFSECVEKHA

>A. lyrata (492640) PTOX

YFAFMSVLHMYETFGWWRRADYLKVHFAESWNEMHHLLIMEELGGNSWWFDRFLAQHIATFYYFMTVFLYILSPRMAYHFSECVESHA

>C. melo (MELO3C021708P2)

YFAFLSVLHLYESFGWWRRADYLKVHFAESWNEMHHLLIMEELGGNDWWFDRFLAQHIAVAYYFMTVFMYMISPRMAYHLSECVESHA

>C. melo (MELO3C021713P1)

YFAFVSVLHMYESFGWWRRADYLKVHFAESWNEMHHLLIMEELGGNDWWFDRFLAQHIAVAYYFMTVFMYMISPRMAYHFSECVESHA

>T. parvula (Tp7g20380)

YFAFMSVLHMYETFGWWRRADYLKVHFAESWNEMHHLLIMEELGGNSWWFDRFLAKNIATFYYFMTVFLYIISPRMAYHFSECVESHA

>M. acuminate (GSMUA_Achr4P11420_001)

YFAFISVLHMYESFGWWRRSDYLKVHFAQSWNEFHHLLIMEELGGNAFWLDRLLAQFVAFFYYFMTVGMYIMSPRMAYHFSECVERHA

>J. curcas (Jcr4S03605.20)

YFAFMSVLHMYESFGWWRRADYLKVHFAESWNEMHHLLIMEELGGNAWWFDRFLAQHIAIFYYVMTVFMYALSPRMAYHFSECVESHA

>C. lanatus (Cla004584)

YFAFLSVLHMYESFGWWRRADYLKVHFAESWNEMHHLLIMEELGGNDWWFDRFLAQHIAVAYYFMTVLMYMISPRMAYHFSECVESHA

>C. grandiflora (Cagra.4739s0006.1)
YFAFMSVLHMYETFGWWRRADYLKVHFAESWNEMHHLLIMEELGGNSWWFDRFLAQHIATFYYFMTVFLYIVSPRMAYHFSECVESHA

>C. mollissima (maker-scaffold10095-augustus-gene-0.12v1)

YFAFMSVLHMYESFGWWRRADYIKVHFAESWNEMHHLLIMEELGGNAWWIDRFLAQHIAVAYYFMTVLMYIISPRMAYHFSECVEGHA

>C. mollissima (maker-scaffold10095-augustus-gene-0.12v2)

YFAFMSVLHMYESFGWWRRADYIKVHFAESWNEMHHLLIMEELGGNAWWIDRFLAQHIAVAYYFMTVLMYIISPRMAYHFSECVEGHA

**AOX**

>Z. mays (GRMZM2G074761_T01)

GMVGGMLLHLRSLRRFEHSGGWIRALLEEAENERMHLMTFLEVAQPKWWERALVLAAQGVYFNAYFVAYLASPKFAHRFVGYLEEEAVHSYTEYLKDLEAGIIDNTPAPAIAIDYWRLPADARLKDVVAVVRADEAHHRD

>Z. mays (GRMZM2G125669_T01)

GMVGGMLLHLRSLRRFEQSGGWIRALLEEAENERMHLMTFMEVAKPRWYERALVITVQGVFFNAYFLGYLLSPKFAHRVVGYLEEEAIHSYTEYLKDLEAGKIENVPAPAIAIDYWRLPANATLKDVVTVVRADEAHHRD

>Z. mays (GRMZM2G074743_T01)

GMVGGMLLHLRSLRRFEHSGGWIRALLEEAENERMHLMTFLEVTQPRWWERALVLTAQGVFFNAYFVGYLLSPKFAHRVVGYLEEEAVHSYTEYLKDLEAGIIDNTPAPAIAIDYWRLPADAKLKDVVTVVRADEAHHRD

>Z. mays (AC233960.1_FGT002)

GMVGGMLLHLRSLRRFEHSGGWIRALLEEAENERMHLMTFMEVAKPKWYERALVLAVQGVFFNAYFLGYLISPKFAHRVVGYLEEEAIHSYTEYLKDLEAGKIENVPAPAIAIDYWQLPADATLKDVVVVVRSDEAHHRD

>D. carota AOX2b (ABZ81230.2)

GMVGGMLLHLKSLRKFQHSGGWIKALLEEAENERMHLMTMVELVQPKWHERLLVLAVQGVFFNAFFVLYILSPKLAHRIVGYLEEEAIHSYTEYLKDIDRGLIENVPAPAISIDYWRLPQDAKLRDVILVIRADEAHHRD

>D. carota AOX2a (ADB24724.1)

GMVGGMLLHLRSLRKFQQSGGWIKALLEEAENERMHLMTMVELVKPKWYERFLVLTVQGVFFNAFFVLYMMSPKVAHRVVGYLEEEAIHSYTEYLKDIESGAIENVPAPAIAIDYWRLPKDAKLKDVITVIRADEAHHRD

>D. carota AOX1 (ABZ81227.2)

GMVGGMLLHCKSLRRFEHSGGWIKTLLDEAENERMHLMTFMEVSQPRWYERALVFTVQGVFFNAYFLAYLASPKLAHRVVGYLEEEAIHSYTEFLKELDKGTIENVPAPAIAIDYWRLPADSTLRDVVMVVRADEAHHRD

>S. bicolor (Sb04g030820.1)

GMVGGMLLHLRSLRRFEHSGGWIRALLEEAENERMHLMTFMEVAKPKWYERALVLAVQGVFFNAYFLGYLISPKFAHRVVGYLEEEAIHSYTEYLKDLEAGKIENVPAPAIAIDYWQLPADATLKDVVVVVRSDEAHHRD

>S. bicolor (Sb06g027410.1)

GMVGGMLLHLRSLRRFEQSGGWIRALLEEAENERMHLMTFMEVAKPRWYERALVITVQGVFFNAYFLGYLLSPKFAHRVVGYLEEEAIHSYTEYLKDLEAGKIENVPAPSIAIDYWRLPANATLKDVVTVVRADEAHHRD

>S. bicolor (Sb06g027420.1)

GMVGGMLLHLGSLRRFEHSGGWIRALLEEAENERMHLMTFLEVAQPKWWERALVLAAQGVFFNAYFVAYLASPKFAHRFVGYLEEEAVHSYTEYLKDLEAGVIENTPAPAIAIDYWRLPADAKLKDVVTVVRADEAHHRD

>S. bicolor (Sb06g027430.1)

GMVGGMLLHLRSLRRFEHSGGWIRALLEEAENERMHLMTFLEVTQPKWWERALVLATQGVFFNAYFVGYLLSPKFAHRVVGYLEEEAVYSYTEYLKDLEAGIIENTPAPAIAIDYWRLPADAKLKDVVTVVRADEAHHRD

>M. guttatus (mgv1a008863m.g) GMVGGMLLHCKSLRRFEHSGGWIKALLEEAENERMHLMTFMEVSQPRWYERALVFTVQGVFFNAYFLTYLISPKMAHRVVGYLEEEAIHSYTEFLKELDKGNIENVPAPAIAIDYWRLPPNSTLRDVVVVVRADEAHHRD

>M. guttatus (mgv1a009239m.g)

GMVGGMLLHAKSLRRFEHSGGWIKALLEEAENERMHLMTFLELSQPQWYERALVFAVQGVFFNAYFVAYVVSPKLAHRIVGYLEEEAVNSYTEFLIDLEKGLVENQPAPAIAIDYWRLPPDATLKDVVTVIRADEAHHRD

>M. guttatus (mgv1a009352m.g)

GMVGGMLLHLRSLRKFQQSGGWIKALLEEAENERMHLMTMVELVQPKWYERFLVLTVQGVFFNAFFTLYLLSPKLAHRVVGYLEEEAIHSYTEYLKDIDDGKIENVRAPAIAIDYWRLPKDATLKDVITVIRADEAHHRD

>A. coerulea (Aquca_033_00110.1)

GMVGGMLLHLRSLRRFQQSGGWIKALLEEAENERMHLMTMLELVKPNWYERALVLTVQGVFFNAFFVLYIISPKLAHRIVGYLEEEAVHSYTEYLEAIETGEIENVPAPAIAIDYWRLPKDATLKDVVIVIRADEAHHRD

>A. coerulea (Aquca_105_00003.1)

GMVGGMLLHCKSLRRFEHSGGWIKALLEEAENERMHLMTFMEVSNPRWYERALVITVQGVFFNAYFLAYMVSPKFAHRVVGYLEEEAIHSYTEFLKELDKGNIENVPAPAIAMDYWRLPPGSTLRDVVTVVRADEAHHRD

>A. coerulea (Aquca_043_00024.1)

GMVGGMLLHCKSLRRFEHSGGWIKALLEEAENERMHLMTFMEVANPRWYERALVFTVQGVFLNAYFLAYMVSPKFAHRVVGYLEEEAIHSYTEFLKELDMGNIKNVPAPAIAMDYWRLPPGSTLRDVVMVVRADEAHHRD

>A. coerulea (Aquca_008_00134.1)

GMVGGMLLHLRSLRRFQQSGGWIKALLEEAENERMHLMTMVELVKPNWYERALVLTVQGVFFNAFFVLYIISPKLAHRIVGYLEEEAVHSYTEYLKDIETGAIENVPAPAIAIDYWRLPKDATLKDVVMVIRADEAHHRD

>B. rapa (Bra031351)

GMVGGMLLHCKSLRRFEQSGGWIKALLEEAENERMHLMTFMEVAKPKWYERALVITVQGVFFNAYFLGYLISPKFAHRMVGYLEEEAIHSYTEFLKELDKGNIENVPAPAIAIDYWRLPADATLRDVVMVVRADEAHHRD

>B. rapa (Bra037768)

GMVGGMLLHLKSIRRFEHSGGWIKALLEEAENERMHLMTMMELVKPKWYERLLVILVQGVFFSSFFTCYVLSPRLAHRIVGYLEEEAIHSYTEFLKDIDDGKIENVKAPAIAIDYWRLPEDATLKDVVTVIRADEAHHRD

>B. rapa (Bra001865)

GMVGGMLLHFKSLRRFEQSGGWIKALLEEAENERMHLMTFMEVAKPKWYERALVITVQGVFFNAYFLGYLISPKFAHRMVGYLEEEAIHSYTEFLKELDKGNIDNVPAPAIAIDYWRLPADATLRDVVMVVRADEAHHRD

>B. rapa (Bra023835)

GMVGGMLVHCKSLRRFEQSGGWIKALLEEAENERMHLMTFMEVAKPNWYERALVIAVQGVFFNAYFLGYIISPKFAHRMVGYLEEEAIHSYTEFLKELDNGNIENVPAPAIAIDYWRLPADATLRDVVMVVRADEAHHRD

>B. rapa (Bra010153)

GMVGGMLLHLKSLRRFEHSGGWIKALLEEAENERMHLMTFIELSQPKWYERAIVFAVQGVFFNAYFLSYVVSPKLAHRITGYLEEEAVNSYTEFLNDIDAGKFKNSPAPAIAIDYWRLPKDATLRDVVFVIRADEAHHRD

>G. aurea AOX (EPS63444.1)

GMVAGMLLHCKSLRRFEHSGGWIKALLEEAENERMHLMTFMEVSQPRWYERALVFAVQGVFFNAYLLAYLISPKLAHRVVGYLEEEAIHSYTEFLKELDKGTIENVPAPAIALDYWRMEPGSTLRDVVMVVRADEAHHRD

>O. europaea AOX1c (JX912721.1)

GMVGGMLLHLKSIRRFEHSGGWIKALLEEAENERMHLMTFLELSQPKWYQRALVFAVQGVFANAYFVSYVVSPKLAHRIVGYLEEEAVNSYTEFLIDLEKGLVENRPAPAIAIDYWQLPSESTLKDVVTVIRADEAHHRD

> O. europaea AOX2 partial (JX912722.1)

GMVGGMLLHLRSLRKFEQSGGWIKALLEEAENERMHLMTMVELVQPKWYERVLVLTVQGVFFNAFFVLYLLSPKLAHRVVGYLEEEAIHSYTEYLKDIDSGKIENVPAPAIAIDYWRLPKDATLKDVITVIRADEAHHRD

>O. europaea AOX1a (accession)

GMIGGMLLHCKSLRRFEHSGGWIKALLEEAENERMHLMTFMEVSQPRWYERALVFTVQGVFFNAYFLTYLVSPKLAHRVVGYLEEEAIHSYTEFLKELDKGTIENVPAPAIAIDYWRMPPNSTLRDVVMVVRADEAHHRD

>Pavir.Gb00789.1
GMVGGMLLHLRSLRRFEQSGGWIRALMEEAENERMHLMTFMEVAKPRWYERALVIAVQGVFFNAYFLGYLLSPKFAHRVVGYLEEEAIHSYTEYLKDLEAGKIDNVPAPAIAIDYWRLPANATLKAVVTVVRADEAHHRD

>Pavir.Gb00785.1
GMVGGMLLHLRSLRRFEHSGGWIRALLEEAENERMHLMTFLEVTQPRWWERALVLAAQGTFFNAYFVGYLLSPKFAHRVVGYLEEEAVHSYTEYLKDLEAGVIENTPAPAIAIDYWGLPADARLKDVVTVVRADEAHHRD

>Pavir.Ab02811.1
GMVGGMLLHLRSLRRFEHSGGWIRVLLEEAENERMHLMTFMEVAKPRWYERALVLAVQGVFFNAYFLGYLISPKFAHRVVGYLEEEAIHSYTEYLRDIEDGKIENVPAPAIAIDYWQLPADATLKDVVTMVRADEAHHRD

>Pavir.Ga00625.1
GMVGGMLLHLRSLRRFEHSGGWIRALLEEAENERMHLMTFLEVTQPRWWERALVLAAQGAFFNAYFVGYLVSPKFAHRVVGYLEEEAVHSYTEYLKDLEAGVIDNTPAPAIAIDYWRLPADARLKDVVTVVRADEAHHRD

>Pavir.Ga00729.1
GMVGGMLLHLRSLRRFEHSGGWIRALLEEAENERMHLMTFLEVAQPKWWERALVLAAQGVYFNAYFVAYLASPKFAHRFVGYLEEEAVHSYTEYLKDLEAGIIENTPAPAIAIDYWRLPADARLKDVVAVVRADEAHHRD

>Pavir.Gb00786.1
GMVGGMLLHLRSLRRFEHSGGWIRALLEEAENERMHLMTFLEVAQPKWWERALVLAAQGVYFNAYFVAYLASPKFAHRFVGYLEEEAVHSYSEYLKDLEAGIIENTPAPAIAIDYWRLPADARLKDVVAVVRADEAHHRD

>P. Virgatum (Pavir.Ab01160.1)
GMVAGAVLHLRSLRRFEQSGGWIRALLEEAENERMHLMTFMEVSQPRWHERALVVAVQGVFLHAYLAAYLLSPATAHRMVGYLEEEAVHSYTEFLRDIDAGKIEDVPAPAIAIDYWRLPAGATLRDVVKVVRADEAHHRD

>P. Virgatum (Pavir.Aa00784.1)
GMVGGMLLHLRSLRRFEHSGGWIRVLLEEAENERMHLMTFMEVAKPRWYERALVLAVQGVFFNAYFLGYLISPKFAHRVVGYLEEEAIHSYTEYLKDIEDGKIENVPAPAIAIDYWQLPADATLKDVVTMVRADEAHHRD

>P. Virgatum (Pavir.Ga00730.1)
GMVGGMLLHLRSLRRFEQSGGWIRALLEEAENERMHLMTFMEVAKPRWYERALVIAVQGVFFNAYFLGYLLSPKFAHRVVGYLEEEAIHSYTEYLKDLEVGKIDNVPAPAIAIDYWRLPANATLKDVVTVVRADEAHHRD

>S. lycopersicum (XP_004230603.1)

GMVGGMLLHLRSLRKFEHSGGWIKALLEEAENERMHLMTMVELVQPKWYERLLVIAVQGVFFNFYSVLYLLSPKLAHRVVGYLEEEAIHSYTLYLNDIDRGEIENVPAPAIAIDYWRLPKDATLKDVITVIRADEAHHRD

>S. lycopersicum AOX1a (NP_001234117.1)

GMVGGMLLHCKSLRRFEQSGGWIKALLEEAENERMHLMTFMEVAKPNVYERALVFAVQGVFFNAYFAAYLISPKLAHRIVGYLEEEAVHSYTEFLKELDNGNIENVPAPAIAIDYWRLPKDATLRDVVLVVRADEAHHRD

>M. esculenta (cassava4.1_010822m.g)

GMVAGMLLHLRSLRRFQQSGGWIKALLEEAENERMHLMTMVELVQPRWYERFLVLAVQGVFFNAYFVLYLLSPKLAHRITGYLEEEAIHSYTEFLKDIKNGQIENVPAPAIAIDYWRLPNDATLEDVITVIRADEAHHRD

>S. Lycopersicum AOX1b (NP_001234120.1)

GMVGGMLLHCKSLRRFEHSGGWIKALLEEAENERMHLMTFIELSNPKWYERALVFAVQGVFVNAYFIAYLASPKLAHRIVGYLEEEAVNSYTEFLIDIEKGLFENSPAPAIAIDYWRLPADATLKDVVTVIRADEAHHRD

>S. lycopersicum (Solyc08g005550.2.1)

GMVGGMLLHCKSLRRFEHSGGWIKALLEEAENERMHLMTFMEVSKPKWYERGLVLMVQGIFFNVYFMTYILSPKLAHRIVGYLEEEAIHSYTQFLKELDEGNIENVAAPAIAIDYWRLTQDATLKDVVMVVRADEAHHRD

>A. thaliana AOX1c (NM_113678.2)

GMVGGMLMHFKSLRRFEQSGGWIKALLEEAENERMHLMTFMEVAKPKWYERALVISVQGVFFNAYLIGYIISPKFAHRMVGYLEEEAIHSYTEFLKELDNGNIENVPAPAIAVDYWRLEADATLRDVVMVVRADEAHHRD

>O. europaea (ABZ81228.2, old carrot)

GMVGGMLLHCKSLRRFEHSGGWIKALLEEAENERMHLMTFMEVSQPRWYERALVFTVQGVFFNAYFLTYLVSPKLAHRVVGYLEEEAIHSYTEFLKELDKGTIENVPAPAIAIDYWRMPPNSTLRDVVMVVRADEAHHRD

>A. thaliana AOX1b (NM_113134.1)

GMVGGMLVHCKSLRRFEQSGGWIKALLEEAENERMHLMTFMEVAKPNWYERALVIAVQGIFFNAYFLGYLISPKFAHRMVGYLEEEAIHSYTEFLKELDNGNIENVPAPAIAIDYWRLEADATLRDVVMVVRADEAHHRD

>A. thaliana AOX2 (NM_125817.2)

GMVGGMLLHLKSIRKFEHSGGWIKALLEEAENERMHLMTMMELVKPKWYERLLVMLVQGIFFNSFFVCYVISPRLAHRVVGYLEEEAIHSYTEFLKDIDNGKIENVAAPAIAIDYWRLPKDATLKDVVTVIRADEAHHRD

>A. thaliana AOX1d (NP_564395.1)

GMVGGMLLHLKSLRRFEHSGGWIKALLEEAENERMHLMTFIELSQPKWYERAIVFTVQGVFFNAYFLAYVISPKLAHRITGYLEEEAVNSYTEFLKDIDAGKFENSPAPAIAIDYWRLPKDATLRDVVYVIRADEAHHRD

>A. thaliana AOX1a (NM_113135.3)

MVGGMLLHCKSLRRFEQSGGWIKALLEEAENERMHLMTFMEVAKPKWYERALVITVQGVFFNAYFLGYLISPKFAHRMVGYLEEEAIHSYTEFLKELDKGNIENVPAPAIAIDYWRLPADATLRDVVMVVRADEAHHRDV

>S. tuberosum (PGSC0003DMT400032705)

GMVGGMLLHLRSLRKFEQSGGWIKALLEEAENERMHLMTMVELVQPKWYERLLVIAVQGVFFNFYFVLYLLSPKLAHRVVGYLEEEAIHSYTLYLNDIDRGEIENVPAPAIAIDYWRLPKDATLKDVITVIRADEAHHRD

>S. tuberosum (PGSC0003DMT400019708)

GMVGGMLLHCKSLRRFEQSGGWIKALLEEAENERMHLMTFMEVAKPNVYERALVFAVQGVFFNAYFAAYLISPKLAHRIVGYLEEEAVHSYTEFLKELDNGNIENVPAPAIAIDYWRLPKDATLRDVVLVVRADEAHHRD

> S. tuberosum (Sotub08g008380.1.1)

GMVGGMLLHCKSLRRFEHSGGWIKALLEEAENERMHLMTFMEVSKPKWYERALVLIIQGIFFNVYFMTYILSPKLAHRIVGYLEEEAIHSYTQFLKELDEGNIENVAAPAIAIDYWRLTQEATLKDVVMVVRADEAHHRD

>S. tuberosum AOX (2208475A)

GMVGGLLLHLKSLRRFEHSGGWIKALLEEAENERMHLMTFMEVSQPRWYERALVLAVQGVFFNAYFLGYLLSPKFAHRVVGYLEEEAIHSYTEFLKEIDKGTIDNVPAPAIALDYWRLPPGSTLRDVVMVVRADEAHHRD

>S. tuberosum AOX (AB176953.1)

GMVGGMLLHCKSLRRFEQSGGWIKALLEEAENERMHLMTFMEVAKPNVYERALVFAVQGVFFNAYFAAYLISPKLAHRIVGYLEEEAVHSYTEFLKELDNGNIENVPAPAIAIDYWRLPKDATLRDVVLVVRADEAHHRE

> S. tuberosum (PGSC0003DMT400019707) GMVGGMLLHCKSLRRFEHSGGWIKALLEEAENERMHLMTFIELSNPKWYERALVFAVQGVFVNAYFLAYLASPKLAHRIVGYLEEEAVNSYTEFLIDIEKGLFENSPAPAIAIDYWRLPADATLKDVVTVIRADEAHHRD

>S. tuberosum AOX1a (ABB76768.1)

GMVGGMLLHCKSLRRFEQSGGWIKALLEEAENERMHLMTFMEVAKPNVYERALVFTVQGVFFNAYFAAYLISPKLAHRIVGYLEEEAVHSYTEFLKELDNGNIENVPAPAIAIDYWRLPKDATLRDVVLVVRADEAHHRD

>S. tuberosum (PGSC0003DMT400047562)

GMVGGMLLHCKSLRRFEHSGGWIKALLEEAENERMHLMTFMEVSKPKWYERALVLIIQGIFFNVYFMTYILSPKLAHRIVGYLEEEAIHSYTQFLKELDEGNIENVAAPAIAIDYWRLTQEATLKDVVMVVRADEAHHRD

>M. sativa AOX1 (AGQ42774.1)

GMVGGMLLHCKSLRRFEHSGGWIKALLEEAENERMHLMTFMEVAKPKWYERALVITVQGVFFNAYFLGYLLSPKFAHRMVGYLEEEAIHSYTEFLKELDKGNIENVPAPAIAIDYWQLPQNSTLRDVVEVVRADEAHHRD

>M. sativa AOX2a (AGQ42775.1)

GMVGGMLLHLKSLRKFQHSGGWVKALLEEAENERMHLMTMVELVKPKWYERFLVLAVQGVFFNAFFVLYILSPKVAHRVVGYLEEEAIHSYTEYLKDIDSGAIENVPAPAIAIDYWRLPKDAKLKDVITVIRADEAHHRD

>T. cacao AOX1b (EOY25435.1)

GMVGGMLLHCKSLRGFEHSGGWIKALLEEAENERMHLMTFMEVAKPRWYDRALVLAVQGVFFNAYFLGYIISPKFAHRMVGYLEEEAIHSYTEFLKELDNGNIENVPAPAIAIDYWRLAPDSTLRDVVMVVRADEAHHRD

>T. cacao AOX2 (EOX98793.1)

GMVGGMLLHLRSLRKFQHSGGWIKALLEEAENERMHLMTMVELVKPKWYERLLVLTVQGVFFNAFFVLYVLSPKLAHRIVGYLEEEAIHSYTEYLKDIESGAIENVPAPAIAIDYWRLPKDANLKDVITVIRADEAHHRD

>P. tremula x P. tremuloides AOX (CAB64356.1)

GMVGGMLLHCKSLRRFEHSGGWIKTLLDEAENERMHLMTFMEVAKPRWYERALVITVQGVFLNAYFLGYIISPKFAHRMVGYLEEEAIHSYTEFLKELDKGNIENVPAPAIAVDYWRLPPDATLRDVVLVVRADEAHHRD

>N. attenuata AOX (AAR37365.1)

GMVGGMLLHCKSLRRFEQSGGWIKALLEEAENERMHLMTFMEVAKPNWYERALVFAVQGVFFNAYFVTYLVSPKLAHRIVGYLEEEAIHSYTEFLKELDKGNIENVPAPAIAIDYWRLPKDSTLRDVVVVVRADEAHHRD

>N. attenuata (AAR37364.1)

GMVGGMLLHCKSLRRFEQSGGWIKALLEEAENERMHLMTFMEVAKPNWYERALVFAVQGVFFNAYFVTYLVSPKLAHRIVGYLEEEAIHSYTEFLKELDKGNIENVPAPAIAIDYWRLPKDSTLRDVVLVVRADEAHHRD

>A. maculatum AOX1f (BAJ22113.1)

GMVGGLLLHLKSLRRFEHSGGWIKTLLEEAENERMHLMTFMEVSQPRWYERALVLAVQGVFFNAYFLGYLISPKFAHRVVGYLEEEAIHSYTEFLKEIDKGTIENVPAPAIALDYWRLPPGSTLRDVVMVVRADEAHHRD

> A. maculatum AOX1d (BAJ22111.1)

GMVGGVLLHLKSLRRFDHSGGWIKALLEEAENERMHLMTFMEVSQPRWYERALVLAVQGVFFNAYFLGYLISPKFAHRVVGYLEEEAIHSYTEFLKEIDKGTIENVPAPAIALDYWRLPPGSTLRDVVMVVRADEAHHRD

>A. maculatum AOX1 c (BAJ22110.1)

GMVGGLLLHLKSLRRFEHSGGWIKTLLEEAENERMHLMTFMEVSQPRWYERALVLAVQGVFFNAYFLGYLISPKFAHRVVGYLEEEAIHSYTEFLKEIDKGTIENVPAPAIALDYWRLPPGSTLRDVVMVVRADEAHHRD

>G. max (Glyma05g24455.1)

GMVGGMLLHLRSLRKFQPSGGWIKALLEEAENERMHLMTMVELVKPKWYERLLVLAVQGVFFNAFFVLYILSPKVAHRIVGYLEEEAIHSYTEYLKDLESGAIENVPAPAIAIDYWRLPKDAKLKDVVTVIRADEAHHRD

>G. max AOX2a (AAP68984.1)

GMVGGMLLHLRSLRKFQQSGGWIKALLEEAENERMHLMTMVELVKPKWYERLLVLAVQGVFFNAFFVLYILSPKVAHRIVGYLEEEAIHSYTEYLKDLESGAIENVPAPAIAIDYWRLPKDARLKDVITVIRADEAHHRD

>G. max (Glyma04g14800.1)

GMVAGMLLHCKSLRRFEHSGGWIKALLEEAENERMHLMTFMEVAKPKWYERALVITVQGVFFNAYFLGYLLSPKFAHRMVGYLEEEAIHSYTEFLKELDKGNIENVPAPAIAIDYWQLPPGSTLRDVVMVVRADEAHHRD

>G. max (Glyma08g07690.1)

GMVGGMLLHLKSLRKFQHSGGWIKALLEEAENERMHLMTMVELVKPSWHERLLIFTAQGVFFNAFFVFYLLSPKAAHRFVGYLEEEAVISYTQHLNAIESGKVENVPAPAIAIDYWRLPKDATLKDVVTVIRADEAHHRD

>C. sativus AOX2 (AAP33163.2)

GMVGGMLLHLKSLRKFQHSGGWIKALLEEAENERMHLMTMIELVQPKWYERLLVITVQGVFFNAFFVLYLMSPKLAHRIVGYLEEEAIHSYTEYLKDINEGKIENVPAPAIAIDYWRLPKDARLKDVITVIRADEAHHRD

>N. tabacum (AAC60576.1)

GMVGGMLLHCKSLRRFEQSGGWIKTLLDEAENERMHLMTFMEVAKPNWYERALVFAVQGVFFNAYFVTYLLSPKLAHRIVGYLEEEAIHSYTEFLKELDKGNIENVPAPAIAIDYCRLPKDSTLLDVVLVVRADEAHHRD

>N. Glutinosa (ABU24346.1)

GMVGGMLLHCKSLRRFEQSGGWIKALLEEAENERMHLMTFMEVAKPNWYERALVFAVQGVFFNAYFVTYLLSPKLAHRIVGYLEEEAIHSYTEFLKELDKGNIENVPAPAIAIDYWRLPKDSTLRDVVLVVRADEAHHRD

>P. patens (Pp1s183_11V6.3)

GMVGGMLLHCKSLRKFQNSGGWIKALLEEAENERMHLMTFMEVAQPKWWERALVFAVQGVFFNAYFLLYLVSPKIAHRITGYLEEEAVYSYTQFLKMIDEGHFQNGPAPSIAIDYWRLPKDATIRDVVMVVRADEAHHRD

>B. distachyon (Bradi5g20540.1)

GMVGGMLLHLRSLRRFEQSGGWIRALLEEAENERMHLMTFMEVAQPRWYERALVIAVQGVFFNAYFFGYLISPKFAHRVVGYLEEEAVHSYTEFLKDLEAGKIDDVPAPSIAIDYWRLPANATLKDVVTVVRADEAHHRD

>B. distachyon (Bradi3g52505.1)

GMVGGMLLHLRSLRRFEHSGGWIRALLEEAENERMHLMTFMEVAGPKWYERALVLAVQGVFFNAYFLGYLLSPKFAHRVVGYLEEEAVHSYTEFLRDIEAGKIDNVPAPRIAIDYWRLPPDATLRDVVVVVRADEAHHRD

>B. distachyon (Bradi5g20557.1)

PMVGGMLLHLRSLRRFEHSGGWIRALMEEAENERMHLMTFLEVTQPNWWERALVMAAQGVFVNAYFVGYLVSPKFAHRFVGYLEEEAVHSYTEYLKDLEAGKIENTPAPAIAIDYWRLPADATLKDVVTVIRADEAHHRD

>B. distachyon (Bradi5g20547.1)

PMVGGMLLHLRSLRRFEHSGGWIRALMEEAENERMHLMTFLEVTQPKWWERALVMAVQGVFFNAYFVGYLVSPKFAHRFVGYLEEEAVKSYTEYLKDLEAGKIENTPAPAIAIDYWRLPADATLKDVVAVVRADEAHHRD

>S. moellendorffii (15407592_peptide)

GMVGGMLLHCRSLRRFEHSGGWIKALLEEAENERMHLMTFMEVVQPKWYERALVFAVQGVFFNAYMVCYIAFPRLAHRIVGYLEEEAIHSYTEYIKEIDKGNIPNTPAPAIAIDYWRLPKDAKIRDVVQVVRADEAHHRD

>S. moellendorffii (15415147_peptide)

GMVGGMLLHCKSLRKFEHSGGWIKALLEEAENERMHLMTFMEVFQPKWYERALVLAVQGVFFNGYFALYLIFPRLAHRFVGYLEEEAVASYTEFLAALDKGSMPNTPAPAIAIDYWRLPSDATLRDVVFVVRADEAHHRD

>S. moellendorffii (15416501_peptide)

GMVGGMLLHCKSLRRFEHSGGWIKALLEEAENERMHLMTFMEVVQPKWYERALVFTVQGVFFNAYFLCYVLFPRLAHRIVGYLEEEAIHSYTEYLKEIDKGTIPNAPAPAIAIDYWRLPKDAKMRDVVEVVRADEAHHRD

>S. moellendorffii (15417125_peptide)

GMVGGMLLHCKSLRRFEHSGGWIKALLEEAENERMHLMTFMEVVQPKWYERALVFTVQGVFFNAYFLCYVLFPRLAHRIVGYLEEEAIHSYTQYLKEIDKGTIPNVPAPAIAIDYWRLPKDAKMRDVVEVVRADEAHHRD

>R. communis (30063.t000003)

GMVGGMLLHLRSLRKFQQSGGWIKALLEEAENERMHLMTMVELVQPRWYERLLVLAVQGVFFNAYFVLYLLSPKLAHRITGYLEEEAIHSYTEFLKDIKEGKIENIPAPAISIDYWRLPKDATLEDVITVIRADEAHHRD

>V. vinifera (GSVIVT01038745001)

GMVGGMLLHLRSLRKFEHSGGWVKALLEEAENERMHLMTMVELVRPKWYERLLVLTVQGVFFNAFFVLYVLSPKAAHRVVGYLEEEAIHSYTEFLKDIDSGAIENVPAPAIAIDYWRLPKDATLKDVITVIRADEAHHRD

>V. vinifera (GSVIVT01022814001)

GMVGGMLLHCKSLRRFEHSGGWIRVLLEEAENERMHLMTFMEVAKPRWYERALVFAVQGIFWNFYFVAYVISPKVAHRAVGYLEEEAIHSYNEFIKELDSGNIPNVPAPAIAIDYWRLAPDSTLRDVVMVVRADEAHHRD

>V. vinifera AOX2 (NP_001268001.1)

GMVGGMLLHLRSLRKFEHSGGWVKALLEEAENERMHLMTMVELVRPKWYERLLVLTVQGVFFNAFFVLYVLSPKAAHRVVGYLEEEAIHSYTEFLKDIDSGAIENVPAPAIAIDYWRLPKDATLKDVITVIRADEAHHRD

>V. vinifera AOX3 (XP_002262982.1)

GMVGGMLLHCQSPRRFEQSGGWIKALLEEAENERMHLMTFIELAKPQWYERAIVFAVQGVFFNAYFLTYLASPKVAHRITGYLEEEAVRSYTEFLKDLDNGSFENVPAPAIAIDYWRLPAESTLRDVVEVIRADEAHHRD

>M. domestica (MDP0000643331)

GMVGGMLLHLRSLRKFQHSGGWIKALLEEAENERMHLMTMVELVKPVWYERLLVLXVQGVFFNAFFVLYMISPKLAHRIVGYLEEEAIHSYTEYLKDIDSGKIENVQAPAIAIDYWRLPKDAKLKDVITVIRADEAHHRD

>M. domestica (MDP0000323076) GMVGGMLLHLRSLRKFQHSGGWIKALLEEAENERMHLMTMVELVKPVWYERLLVLXVQGVFFNAFFVLYMISPKLAHRIVGYLEEEAIHSYTEYLKDIDSGKIENVQAPAIAIDYWRLPKDAKLKDVITVIRADEAHHRD

>M. domestica (MDP0000244591)

GMVGGMLLHLKSLRKFEHSGGWIKALLEEAENERMHLMTVVELVKPAWHERLLVLAVQGVFFNAFFGLYLASPKLAHRVVGYLEEEAVFSYTEYLKDIENGKIENVKAPEIAIDYWRLPKGATLHDVITVIRADEAHHRD

>M. domestica (MDP0000244591)

GMVGGMLLHLKSLRKFEHSGGWIKALLEEAENERMHLMTVVELVKPAWHERLLVLAVQGVFFNAFFGLYLASPKLAHRVVGYLEEEAVFSYTEYLKDIENGKIENVKAPEIAIDYWRLPKGATLHDVITVIRADEAHHRD

>P. trichocarpa (Potri.015G019800.1)

GMVGGMLLHCKSLRRFEHSGGWIKTLLDEAENERMHLMTFMEVAKPRWYERALVITVQGVFLNAYFLGYIISPKFAHRMVGYLEEEAIHSYTEFLKELDKGNIENVPAPAIAVDYWRLPPNATLRDVVLVVRADEAHHRD

>P. trichocarpa (Potri.012G001500.1)

GMVGGMLLHCKSLRRFEHSGGWIKALLEEAENERMHLMTFMEVANPRWYERALVITVQGVFFNAYFLGYLISPKFAHRMVGYLEEEAIHSYTEFLKELDKGNIKNVPAPAIAIDYWRLPPNSTLRDVVEVVRADEAHHRD

>P. trichocarpa (Potri.012G001600.1)

GMVGGMLLHCRSLRRFEHSGGWIKALLEEAENERMHLMTFMEVSNPRWYERALVFTVQGVFFNAYFLGYLISPKFAHRMVGYLEEEAIHSYTEFLKELDKGNIKNVPAPAIAIDYWRLPPNSTLRDVVVAVRADEAHHRD

>P. trichocarpa (Potri.003G103900.1)

GMVGGMLLHCKSLRRFEQSGGWIKALLEEAENERMHLMTFVEIAKPQWYERALVFAVQGAFFNAYFLAYLASPKLAHRIVGYLEEEAVNSYSEFLEDLDNGNFENVPAPAIAIDYWRLPPNSTLRDVVFVIRADEAHHRD

>P. vulgaris (Phvul.002G209200.1)

GMVGGMLLHLKSLRKFQHSGGWIKALLEEAENERMHLMTMVELVNPKWHERLLIFAAQGVFFNGFFVFYLLSPKAAHRFVGYLEEEAVISYTQHLEAIESGKVENVPAPAIAIDYWRLPKDATLKDVVTVIRADEAHHRD

>P. vulgaris (Phvul.002G209100.1) GMVGGMLLHLRSLRKFQQSGGWIKALMEEAENERMHLMTMVELVKPKWYERLLVLAVQGVFFNAFFALYILSPKVAHRIVGYLEEEAIHSYTEYLKDIERGAIENVPAPAIAIDYWRLPKDAKLKDVITVIRADEAHHRD

>P. vulgaris (Phvul.002G127100.1)

GMVGGMLLHFKSLRRFEQSGGWIKALLEEAENERMHLMTFMEVAKPKWYERALVITVQGVFFNAYFLGYMISPKFAHRMVGYLEEEAIHSYTEFLKELDKGNIQNVPAPAIAIDYWQLPPDATLRDVVMVVRADEAHHRD

>F. vesca (mrna24637.1-v1.0-hybrid)

GMVGGMLLHCKSLRRFEHSGGWIKALLEEAENERMHLMTFMEVAKPRWYERALVFAVQGVFFNAYFLGYMISPKFAHRMVGYLEEEAIHSYTEFLKELDKGNIENVPAPAIAIDYWQLPPNSTLRDVVTVVRADEAHHRD

>F. vesca (mrna12195.1-v1.0-hybrid)

GMVGGMLLHLRSLRKFQQSGGWIKALLEEAENERMHLMTMVELVKPAWYERLLVLTVQGVFFNAFFGLYMISPKVAHRVVGYLEEEAIHSYTEYLKDIDSGKIENVPAPAIAIDYWRLPKDAKLKDVITVIRADEAHHRD

>F. vesca (mrna12197.1-v1.0-hybrid)

GMVGGMVLHLRSLRRFEHSGGWIKALLEEAENERMHLMTVGELVKPVWHERLLVLAAQGVFFNAFFVFYLLSPKIAHRFTGYLEEEAVISYTDYLKAIEDGKIENVPAPAIAIDYWRLPKDARLLDVITVIRADEAHHRD

>A. lyrata (fgenesh1_pg.C_scaffold_3002143)

GMVGGMLVHCKSLRRFEQSGGWIKALLEEAENERMHLMTFMEVAKPNWYERALVIAVQGVFFNAYFLGYLISPKFAHRMVGYLEEEAIHSYTEFLKELDNGNIENVPAPAIAIDYWRLEADATLRDVVMVVRADEAHHRD

>A. lyrata (fgenesh2_kg.3__2450__AT3G22370.1)

GMVGGMLLHCKSLRRFEQSGGWIKALLEEAENERMHLMTFMEVAKPKWYERALVITVQGVFFNAYFLGYLISPKFAHRMVGYLEEEAIHSYTEFLKELDKGNIENVPAPAIAIDYWRLPADATLRDVVIVVRADEAHHRD

>A. lyrata (fgenesh1_pm.C_scaffold_1002587)

GMVGGMLLHLKSLRRFEHSGGWIKALLEEAENERMHLMTFIELSQPKWYERAIVFTVQGAFFNAYFLAYVISPKLAHRITGYLEEEAVNSYTEFLKDIDAGKFENSPAPAIAIDYWRLPKDATLRDVVYVIRADEAHHRD

>C. papaya (evm.model.supercontig_8.29)

GMVGGMLLHCKSLRKFEHSGGWIKALLEEAENERMHLMTFMEVAKPKWYERALVFAVQGVFFNAYFLGYLVSPKFAHRMVGYLEEEAIHSYTEFLKEIDNGNIENVPAPAIAIDYWRLPSGSTLRDVVMVVRADEAHHRD

>C. papaya (evm.TU.supercontig_42.47)

GMVGGMLLHLKSLRKFQQSGGWIKALLEEAENERMHLMTMVELVQPKWYERLLVLAVQGVFFNAFFVLYILSPKLAHRIVGYLEEEAIHSYTEYLKDIDSGAIKNVPAPAIAIDYWRLPKDATLKDVITVIRADEAHHRD

>C. sinensis (orange1.1g019765m.g)

GMVGGMLLHCKSLRRFEHSGGWIKALLEEAENERMHLMTFMEVAKPKWYERALVFAVQGVFFNAYFLGYLISPKFAHRMVGYLEEEAIHSYTEFLKELDKGNIENVPAPAIATDYWRLPPNSTLKDVVLVVRADEAHHRD

>C. sinensis (orange1.1g020532m.g)

GMVGGMLLHLKSLRKFQHSGGWIKALLEEAENERMHLMTMVELVKPKWYERMLVLTVQGVFFNAFFVLYLLSPKLAHRVVGYLEEEAIHSYTEYLKDIDSGSIENVPAPAIAIDYWRLPKDATLKDVITVIRADEAHHRD

>S. italica (Si010540m.g)

GMVGGMLLHLRSLRRFEQSGGWIRALLEEAENERMHLMTFMEVAKPRWYERALVITVQGVFFNAYFLGYLLSPKFAHRVVGYLEEEAIHSYTEYLKDLEAGKIDNVPAPAIAIDYWRLPANATLKDVVTVVRADEAHHRD

>S. italica (Si010566m.g)

GMVGGMLLHLRSLRRFEHSGGWIRALLEEAENERMHLMTFLEVAQPRWWERALVLAAQGVYFNAYFVAYLASPKFAHRFVGYLEEEAVHSYTEYLKDLEAGLIENTPAPAIAIDYWRLPADARLKDVVTVVRADEAHHRD

>S. italica (Si017726m.g)

GMVGGMLLHLRSLRRFEHSGGWIRALMEEAENERMHLMTFMEVAKPRWYERALVLAVQGVFFNAYFLGYLVSPKFAHRVVGYLEEEAIHSYTEFLKDIEAGKIENVPAPAIAIDYWQLPADARLKDVVTVVRADEAHHRD

>S. italica (Si010539m.g)

GMVGGMLLHLRSLRRFEHSGGWIRALLEEAENERMHLMTFLEVTQPRWWERALVLAAQGVFFNAYFVGYLLSPKFAHRVVGYLEEEAVHSYTEYLKDLEAGVIDNTPAPAIAIDYWRLPADAKLKDVVTVVRADEAHHRD

>T. halophila (Thhalv10005697m.g)

GMVGGMLLHLKSIRRFEHSGGWIKALLEEAENERMHLMTMMELVKPVWHERLLVMLVQGVFFNCFFICYVVSPRLAHRIVGYLEEEAIHSYTEFLKDIDDGKIENVAAPAIAIDYWRLPKDAKLKDVVTVIRADEAHHRD

>T. halophila (Thhalv10009342m.g)

GMVGGMLLHLKSLRRFEHSAGWIKALLEEAENERMHLMTFIELSQPKWYERAIVFAVQGVFFNAYFLSYVISPKLAHRITGYLEEEAVNSYTEFLKDIDAGKFENSPAPAIAIDYWRLPKDATLRDVVFVIRADEAHHRD

>T. halophile (Thhalv10021013m.g)

GMVGGMLLHCKSLRRFEQSGGWIKALLEEAENERMHLMTFMEVAKPKWYERALVITVQGVFFNAYFLGYLISPKFAHRMVGYLEEEAIHSYTEFLKELDKGNIENVPAPAIAIDYWRLPADATLRDVVMVVRADEAHHRD

>T. halophile (Thhalv10022022m.g) partial

GMVGGMLVHCKSLRRFEQSGGWIKALLEEAENERMHLMTFMEVAKPNWYERALVIAVQGVFFNAYFLGYLISPKFAHRMVGYLEEEAIHSYSEFLKKLDNGNIENVPAPAIAIDYWRLEADATLRDVVMVVRADEAHHRD

>C. clementina (Ciclev10001766m.g)

GMVGGMLLHCKSLRRFEHSGGWIKALLEEAENERMHLMTFMEVAKPKWYERALVFAVQGVFFNAYFLGYLISPKFAHRMVGYLEEEAIHSYTEFLKELDKGNIENVPAPAIATDYWRLPPNSTLKDVVLVVRADEAHHRD

>C. clementina (Ciclev10003687m.g) partial

GMVGGMLLHCKSLRKFEHSGGWIKALLEEAENERMHLMTFIELARPQWYERALVFAVQGVFFNAYFLAYLASPKLAHRIVGYLEEEAVNSYTEFLKDLENGSFENAPAPAIAIDYWRMPPDSTLRDVVVVIRADEAHHRD

>C. clementina (Ciclev10028835m.g|Ciclev10028835m)

GMVGGMLLHLKSLRKFQHSGGWIKALLEEAENERMHLMTMVELVKPKWYERMLVLTVQGVFFNAFFVLYLLSPKLAHRVVGYLEEEAIHSYTEYLKDIDSGSIENVPAPAIAIDYWRLPKDATLKDVITVIRADEAHHRD

>C. rubella (Carubv10019557m.g)

GMVGGMLMHFKSLRRFEQSGGWIKALLEEAENERMHLMTFMEVAKPKWYERALVIAVQGVFFNAYLLGYLISPKFAHRMVGYLEEEAIHSYTEFLKELDNGNIENVPAPAIAIDYWRLEADATLRDVVMVVRADEAHHRD

>C. rubella (Carubv10014065m.g)

GMVGGMLLHCKSLRRFEQSGGWIKALLEEAENERMHLMTFMEVAKPKWYERALVITVQGVFFNAYFLGYLISPKFAHRMVGYLEEEAIHSYTEFLKELDKGNIENVPAPAIAIDYWRLPANATLRDVVMVVRADEAHHRD

>C. rubella (Carubv10026681m.g)

GMVGGMLLHLKSIRKFEHSGGWIKALLEEAENERMHLMTMMELVKPKWYERLLVMLVQGVFFNSFLVCYVISPRLAHRIVGYLEEEAIHSYTEFLKDIDDGKIEDVAAPAIAIDYWRLPKDATLKDVVTVIRADEAHHRD

>C. rubella (Carubv10009739m.g)

GMVGGMLLHLKSLRRFEHSGGWIKALLEEAENERMHLMTFIELSQPKWYERVIVFTVQGVFFNAYFMAYVISPKLAHRITGYLEEEAVNSYTEFLEDIDAGKFENSPAPAIAIDYWRLPKDATLRDVVYVIRADEAHHRD

>E. grandis (Eucgr.E01214.1)

GMVGGMLLHCKSLRRFEHSGGWIKALLEEAENERMHLMTFCEIADPAWYERALVFAVQGVFFNAYFLAYVASPKLAHRIVGYLEEEAVISYTEFLKDLDNGSFENRPAPAIAIDYWRLPKDSTLRDVIVVIRADEAHHRD

>E. grandis (Eucgr.E01213.1) mal annoté, recuperer genomique

GMVGGLLLHCKSLRRFEHSGGWIKALLEEAENERMHLMTFMEVSQPRWYERALVFAVQGVFFNAYFLGYLISPKFAHRVVGYLEEEAIHSYTEFLKELDNGSIENVPAPAIAIDYWQLPEGSTLRDVVLVVRADEAHHRD

>E. grandis (Eucgr.I02663.1)

GMVGGMLLHLRSLRKFEHSGGWVKALLEEAENERMHLMTMVELVKPKWYERLLVLSVQGVFFNAYFVLYLLSPKLAHRVVGYLEEEAIHSYTEYLKDINSGAIENVPAPAIAIDYWRLPKDATLKDVITVIRADEAHHRD

>G. raimondii (Gorai.005G220400.1)

GMVGGMLLHLKSLRKFQQSGGWIKALLEEAENERMHLMTMVELVKPKWYERLLVLTVQGVFFNAFFVLYMLSPKLAHRIVGYLEEEAIHSYTEYLKDIESGAIENVAAPAIAIDYWRLPKDARLKEVITVIRADEAHHRD

>G. raimondii (Gorai.005G220500.1)

GMVGGMLLHLKSLRKFQQSGGWIKALLEEAENERMHLMTMVELVKPKWYERLLVLTVQGVFFNAFFVLYMLSPKLAHRIVGYLEEEAIHSYTEYLKDIDSGAIENVPAPAIAIDYWRLPKDATLKDVITVIRADEAHHRD

>G. raimondii (Gorai.008G296600.1)

GMVGGMLLHCKSLRRFEHSGGWIKALLEEAENERMHLMTFMEVSDPRWYERALVFAVQGVFFNAYFLGYIISPKFAHRVVGYLEEEAIHSYTEFLKELDNGNIENVPAPPIAIDYWRLPPNSTLRDVVLAVRADEAHHRD

>G. raimondii (Gorai.012G142200.1)

GMVGGMLLHLKSLRKFQQSGGWIKALLEEAENERMHLMTIVELVKPKWYERLLVLAVQGVFFNGFFVLYLSSPKLAHRFVGYLEEEAVFSYTEYLESIESGETENVPAPAIAIDYWRLPKDARLKDVITVIRADEAHHRD

>B. juncea AOX1a (AEB00555.1)

GMVGGMLLHCKSLRRFEQSGGWIKALLEEAENERMHLMTFMEVAKPKWYERALVITVQGVFFNAYFLGYLISPKFAHRMVGYLEEEAIHSYTEFLKELDKGNIENVPAPAIAIDYWRLPADATLRDVVMVVRADEAHHRD

>L. usitatissimum (Lus10035670.g)

GMVGGMLLHCKSLRKFEHSGGWIRALLEEAENERMHLMTFMEVAQPKWYERALVMAVQGVFFNAYFLGYIISPKFAHRMVGYLEEEAIHSYTEFLKELDKGNIENVPAPAIAIDYWRLPADATLRDVVVVVRADEAHHRD

>L. usitatissimum (Lus10005372.g)

GMVGGMLLHLRSLRKFQQSGGWIKALIEEAENERMHLMTMVELVQPAWYERLLVLAVQGVFFNAYFVLYIMSPKLAHRVVGYLEEEAIHSYTEYLKDIREGKIENVAAPAIAIDYWRLPKDSTLEDVITVIRADEAHHRD

> L. usitatissimum (Lus10020523)

GMVGGMLLHLRSLRKFQQSGGWIKALIEEAENERMHLMTMVELVQPAWYERLLVLAVQGVFFNAYFVLYIMSPKLAHRVVGYLEEEAIHSYTEYLKDIKAGKIENVAAPAIAIDYWRLPKDSTLEDVITVIRADEAHHRD

>P. persica (ppa008213m.g)

GMVGGMLLHLRSLRKFQQSGGWIKALLEEAENERMHLMTMVELVKPVWYERLLVLAVQGVFFNAFFVLYVLSPKLAHRVVGYLEEEAIHSYTEYLKDIDSGKIENVPAPAIAIDYWRLPKDSTLKDVITVIRADEAHHRD

>P. persica (ppa008350m.g) GMVGGMMLHLKSLRKFEHSGGWIKALLEEAENERMHLMTVVELVKPAWHERLLVLAVQGVFFNAFFVLYALSPKVAHRVVGYLEEEAVHSYTEYLKDIENGKIENVKAPAIAIDYWRLPKDATLHDVITVIRADEAHHRD

>P. Persica (ppa024818m.g) partial

GIVGGMVLHLRSLRKFQQSGGWVKALLEEAENERMHLMTMVELVQPMWYERLLVLVVQGVFFNAFFVLYLLSPKLAHRIVGYLEEEAIHSYTEYLKDIDSGKIENVQAPAIAIDYWRLPKDATLRNVITVIRADEAHHRD

>P. Persica (ppa014817m.g) bizarrev

GMVGGMLLHCKSLRRFEHSGGWIKALLEEAENERMHLMTFMEVAKPKWYERALVVTVQGVFFNAYLLGYLLSPKFAHRMVGYLEEEAIHSYTEFLKELDKGNIENVPAPAIAIDYWQLPPNSTLRDVVTVVRADEAHHRD

>V. unguiculata AOX1 (AAZ09196.1)

GMVAGMLLHLKSLRRFEHSGGWIKALLEEAENERMHLMTFMEVAKPKWYERALVITVQGVFFNAYFLGYMISPKFAHRMVGYLEEEAIHSYTEFLKELDKGNIENVPAPAIAIDYWQLPPDSTLKDVVTVVRADEAHHRD

>N. benthamiana AOX1b (AGZ61935.1)

GMVGGMLLHCKSLRRFEQSGGWIKALLEEAENERMHLMTFMEVAKPNWYERALVFAVQGVFFNAYFVTYLLSPKLAHRIVGYLEEEAIHSYTEFLKELDKGNIENVPAPAIAIDYWRLPKDSTLRDVVLVVRADEAHHRD

>N. bethamiana AOXa (AGZ61934.1)

GMVGGMLLHCKSLRRFEQSGGWIKALLEEAENERMHLMTFMEVAKPNWYERALVFAVQGVFFNAYFVTYLLSPKLAHRIVGYLEEEAIHSYTEFLKELDKGNIENVPAPAIAIDYWRLPKDSTLRDVVLVVRADEAHHRD

>N. nucifera AOX1a (BAH56639.1)

GMVGGMLLHLKSLRRFEHSGGWIKTLLEEAENERMHLMTFMEVSQPKWYERALVVAVQGVFFNTYFLGYLISPRFAHRVVGYLEEEAIHSYTEFLKELDKGNIQNVPAPAIAVDYWQLPPDSTLRDVVMVVRADEAHHRD

>M. truncatula (Medtr5g026620.1)

GMVGGMLLHCKSLRRFEQSGGWIKALLEEAENERMHLMTFMEVAKPKWYERALVITVQGVFFNAYFLGYLLSPKFAHRMVGYLEEEAIHSYTEFLKELDKGNIENVPAPAIAIDYWQLPQNSTLRDVVEVVRADEAHHRD

>M. truncatula (Medtr5g070870.1)

GMVGGMLLHLKSLRKFQHAGGWIKALLEEAENERMHLMTMVELVKPSWHERLLVITAQGVFFNGFFVFYILSPKIAHRFVGYLEEEAVISYTQYLNAIESGKVENVPAPAIAIDYWRLPNDATLKDVVTVIRADEAHHRD

>M. truncatula (Medtr5g070680.1)

PMVGGMLLHLKSLRKFQHTGGWIKALLEEAENERMHLMTMVELVKPSWHERLLVITAQGVFFNAFFVFYILSPKTAHRFVGYLEEEAVISYTQHLNAIESGKVENVPAPAIAIDYWRLPKDATLKDVITVIRADEAHHRD

>O. sativa (LOC_Os04g51160.1)

GMVGGMLLHLRSLRRFEQSGGWIRALLEEAENERMHLMTFLEVMQPRWWERALVLAAQGVFFNAYFVGYLVSPKFAHRFVGYLEEEAVSSYTEYLKDLEAGKIENTPAPAIAIDYWRLPADATLKDVVTVIRADEAHHRD

>O. sativa (LOC_Os04g51150.1)

GMVGGMLLHLRSLRRFEQSGGWIRTLLEEAENERMHLMTFMEVANPKWYERALVITVQGVFFNAYFLGYLLSPKFAHRVVGYLEEEAIHSYTEFLKDLEAGKIDNVPAPAIAIDYWRLPANATLKDVVTVVRADEAHHRD

>O. sativa (LOC_Os02g47200.1)

GMVGGMLLHLRSLRRFEHSGGWIRALLEEAENERMHLMTFMEVAKPRWYERALVLAVQGVFFNAYFLGYLLSPKLAHRVVGYLEEEAIHSYTEYLKDIEAGKIENVPAPPIAIDYWRLPAGATLKDVVVVVRADEAHHRD

>O. sativa (LOC_Os02g21300.1)

GMVAGAVLHLRSLRRFEHSGGWIRALLEEAENERMHLMTFMEVSQPRWYERALVVAVQGAFFNAYLASYLLSPRFAHRIVGYLEEEAVHSYTEFLRDLDAGKIDDVPAPAIAIDYWRLPADATLRDVVMVVRADEAHHRD

>C. lanatus AOX2 (ADD84880.1)

GMVGGMLLHLKSLRKFQHSGGWIKALLEEAENERMHLMTMIELVQPKWYERLLVITVQGVFFNAFFVLYLMSPKLAHRIVGYLEEEAIHSYTEYLKDIDEGKIENVPAPAIAIDYWRLPKDARLKDVITVIRADEAHHRD

>C. melo (MELO3C027020P1)

GMVGGMLLHLKSLRKFQHSGGWIKALLEEAENERMHLMTMIELVQPKWYERLLVITVQGVFFNAFFVLYLMSPKLAHRIVGYLEEEAIHSYTEYLKDINEGKIENVPAPAIAIDYWRLPKDARLKDVITVIRADEAHHRD

>T. parvula (Tp3g20140)

GMVGGMLLHCKSLRRFEQSGGWIKALLEEAENERMHLMTFMEVAKPKWYERALVITVQGVFFNAYFLGYLISPKFAHRMVGYLEEEAIHSYTEFLKELDKGNIENVPAPAIAIDYWRLPAGATLRDVVMVVRADEAHHRD

>T. parvula (Tp3g20130)

GMVGGMLVHCKSLRRFEQTGGWIKALLEEAENERMHLMTFMEVAKPNWYERALVIAVQGVFFNAYFLGYIISPKFAHRMVGYLEEEAIHSYTEFLKELDNGNIKNVPAPAIAIDYWRIDADATLRDVVMVVRADEAHHRD

>T. parvula (Tp2g27270)

GLVGGMLLHLKSIRRFEHSGGWIKALLEEAENERMHLMTMMELVKPKWYERLLVMLVQGIFFNSFFICYVISPRLAHRIVGYLEEEAIHSYTEFLKDIDDGKIENVAAPAIAIDYWRLPKDATLKDVVTVIRADEAHHRD

>T. parvula (Tp1g28550) partial

GMVGGMLLHLKSLRRFEHSGGWIKALLEEAENERMHLMTFIELSQPKWYERAIVFTVQGVFFNAYFLSYVISPKLAHRITGYLEEEAVNSYTEFLKDIDAGKFENSPAPAIAIDYWRLPKDATLRDVVFVIRADEAHHRD

>M. acuminate (GSMUA_Achr6P01170_001)

GMVGGMLLHLRSLRHFEHSGGWIRALLEEAENERMHLMTFMEVSQPRWYERALVFAVQGVFFNAYFAAYLLSPKLAHRMVGYLEEEAIHSYTEFLKDLEAGKIENVPAPAIAIDYWRLPADATLKDVVTVVRADEAHHRD

>M. acuminate (GSMUA_Achr1P27810_001)

GMVGGMLLHLRSLRRFEQSGGWIRSLLEEAENERMHLMTFMEVAQPRWYERALVIAVQGVFFNAYFLGYLVSPRFAHRVTGYLEEEAIHSYTEFLRDLEAGEIDNVPAPAIAIDYWRLPADATLKDVVMVVRADEAHHRD

>M. acuminate (GSMUA_Achr6P01300_001)

GMVGGMLLHLRSLRRFEQSGGWIRALLEEAENERMHLMTFMEVAQPRWYERAIVLAVQGVFFNAYFLGYLVSPKFAHRVTGYLEEEAIHSYTEYLRDLEAGKIDNVPAPSIAIDYWRLPADATLKDVVVVVRADEAHHRD

>M. acuminate (GSMUA_Achr5P03810_001)

GMVGGMLLHLRSLRRFEPSGGWIRVLLEEAENERMHLMTFMEVAQPRWYERALVFAVQGVFFNAYFVAYLLSPKLAHRMVGYLEEEAIHSYTEYLKDLEAGKIENVPAPAIAMDYWRLPADATLKDVVMVVRADEAHHRD

>M. acuminate (GSMUA_Achr1P27800_001)

GMVGGMFLHLRSLRRFEQSGGWIRALLEEAENERMHLMTFMEVAQPRWYERALVIAVQGVFFNAYFLGYLVSPKFAHRVTGYLEEEAIHSYTEFLRDLEAGEIDNVPAPAIAIDYWRLPADATLKDVVMVVRADEAHHRD

>J. curcas (Jcr4S02312.80)

GMVGGMLLHLRSLRRFQQSGGWIKALLEEAENERMHLMTMVELVQPRWYERLLVLAVQGVFFNAYFVLYLLSPKLAHRITGYLEEEAIHSYTEFLKDIKDGKIENVPAPAIAIDYWRLPKDATLEDVITVIRADEAHHRD

>C. lanatus (Cla002470)

GMVGGMLLHLKSLRKFQHSGGWIKALLEEAENERMHLMTMIELVQPKWYERLLVITVQGVFFNAFFVLYLMSPKLAHRIVGYLEEEAIHSYTEYLKDIDEGKIENVPAPAIAIDYWRLPKDARLKDVITVIRADEAHHRD

>P. mume (XM_008239818.1)

GMVGGMLLHCKSLRRFEHSGGWIKALLEEAENERMHLMTFMEVAKPKWYERALVVTVQGVFFNAYMLGYLLSPKFAHRMVGYLEEEAIHSYTEFLKELDKGNIENVPAPAIAIDYWQLPPNSTLRDVVTVVRADEAHHRD

>P. mume (XM_008225757.1)

GIVGGMLLHLRSLRKFQQSGGWVKALLEEAENERMHLMTMVELVQPMWYERLLVLLVQGVFFNAFFVLYLLSPKLAHRIVGYLEEEAIHSYTEYLKDIDSGKIENVQAPAIAIDYWRLPKDATLRDVITVIRADEAHHRD

>P. mume (XM_008225708.1)

GMVGGMLLHLRSLRKFQQSGGWIKALLEEAENERMHLMTMVELVKPVWYERLLVLAVQGVFFNAFFVLYVLSPKLAHRVVGYLEEEAIHSYTEYLKDIDSGKIENVPAPAIAIDYWRLPKDSTLKDVITVIRADEAHHRD

>P. mume (XM_008225707.1)

GMVGGMMLHLKSLRKFEHSSGWIKALLEEAENERMHLMTVMELVKPVWHERFLVLAVQGVFFNAFFVLYALSPKVAHRVVGYLEEEAVQSYTEYLKDIENGNIENVKAPAIAIDYWRLPKDATLHDVITVIRADEAHHRD

>G. gnemon (lcl|UCGgnemon_isotig01461)
GMVGGMLLHCKSLRRFQHSGGWIKALLEEAENERMHLMTFMEVVKPKWYEKALVFAVQGVFFNAYFLMYVLSPKLAHRVVGYLEEEAIHSYTEFLKDIDKGTIPNVPAPAIAIDYWRLPRDAKLRDVVMVVRADEAHHRD

>G. gnemon (lcl|UCGgnemon_isotig01460)
GMVGGMLLHCKSLRRFQHSGGWIKALLEEAENERMHLMTFMEVVKPKWYEKALVFAVQGVFFNAYFLMYVLSPKLAHRVVGYLEEEAIHSYTEFLKDIDKGTIPNVPAPAIAIDYWRLPRDAKLRDVVMVVRADEAHHRD

>G. gnemon (lcl|UCGgnemon_isotig01459)
GMVGGMLLHCKSLRRFQHSGGWIKALLEEAENERMHLMTFMEVVKPNLLERALVFAVQGVFFNAYFLMYVISPKLAHRVVGYLEEEAIHSYTEFLKEIDKGTIPNVPAPLIAIDYWRLPKDAKLRDVVMVVRADEAHHRD

>G. gnemon (lcl|UCGgnemon_isotig01458
GMVGGMLLHCKSLRRFQHSGGWIKALLEEAENERMHLMTFMEVVKPNLLERALVFAVQGVFFNAYFLMYVISPKLAHRVVGYLEEEAIHSYTEFLKEIDKGTIPNVPAPLIAIDYWRLPKDAKLRDVVMVVRADEAHHRD

>P. tadea (lcl|PgdbPtadea_5889)
GMVAGMLLHCKSLRKFQHSGGWIKALLEEAENERMHLMTFMEVAKPRWYERALVFTVQGIFFNAYFLTYILSPKLAHRITGYLEEEAIHSYTEFLKELDKGNIPNVPAPAIAIDYWRLPKDSTLRDVVVVVRADEAHHRD

>P. menziesii (lcl|UCPmenziesii_isotig19868)
GMVGGMLLHCKSLRKFQHSGGWIKALLEEAENERMHLMTFMEVAQPKWYERALVFTVQGVFFNAYFLMYIVSPKLAHRIVGYLEEEAIHSYTEFLKELDKGNIPNVPAPAIAIDYWRLPKDSTLRDVVMVIRADEAHHRD

>P. lambertiana (lcl|UCPlambertiana_isotig23628)
GMVGGMLLHCKSLRKFQHSGGWIKALLEEAENERMHLMTFMEVAKPKWYERALVFTVQGVFFNAYFLMYILSPKLAHRVTGYLEEEAIHSYTEFLKELDKGNIPNVPAPAIAIDYWRLPKDSTLRDVVVVIRADEAHHRD

>P. macrophyllus (lcl|UCPmacrophyllus_isotig09152)
GMVGGMLLHCKSLRRFQHSGGWIKALLEEAENERMHLMTFMEVAKPKWYERALVFTVQGVFFNAYFLLYLMSPKLAHRIVGYLEEEAIHSYTEFIKEIDNGNIPNIPAPAIAIDYWRLPKDSTLRDVVMVVRADEAHHRD

>T. baccata (lcl|UCTbaccata_isotig01282)
GMVGGMLLHLKSLRKFEHSGGWIKALLEEAENERMHLMTMVEVVKPKWYERALVLTVQGVFFNAFFVLYLASPKVAHRMVGYLEEEAIHSYTEFLKELDNGNIENVPAPSIAIDYWRLSKDATLRDVVVVIRADEAHHRD

>A. halleri (Araha.16357s0003.1)
GMVGGMLVHCKSLRRFEQSGGWIKALLEEAENERMHLMTFMEVAKPNWYERALVIAVQGVFFNAYFLGYLISPKFAHRMVGYLEEEAIHSYTEFLKELDNGNIENVPAPAIAIDYWRLEADATLRDVVMVVRADEAHHRD

>A. halleri (Araha.62291s0001.1)
GMVGGMLLHCKSLRRFEQSGGWIKALLEEAENERMHLMTFMEVAKPKWYERALVITVQGVFFNAYFLGYLISPKFAHRMVGYLEEEAIHSYTEFLKELDKGNIDNVPAPAIAIDYWRLPADATLRDVVIVVRADEAHHRD

>A. halleri (Araha.17613s0008.1)
GMVGGMLLHLKSIRKFEHSGGWIKALLEEAENERMHLMTMMELVKPKWYERLLVMLVQGIFFNSFLVCYVISPRLAHRIVGYLEEEAIHSYTEFLKDIDDGKIENVAAPAIAIDYWRLPKDATLKDVVTVIRADEAHHRD

>A. halleri (Araha.63057s0001.1)
GMVGGMLLHLKSLRRFEHSGGWIKALLEEAENERMHLMTFIELSQPKWYERAIVFTVQGVFFNAYFLAYVISPKLAHRITGYLEEEAVNSYTEFLKDIDAGKFENSPAPAIAIDYWRLPKDATLRDVVYVIRADEAHHRD

>C. grandiflora (Cagra.0248s0102.1)
GMVGGMLLHLKSIRKFEHSGGWIKALLEEAENERMHLMTMMELVKPKWYERLLVMLVQGIFFNSFLLCYVISPRLAHRIVGYLEEEAIHSYTEFLKDIDDGKIEDVAAPAIAIDYWRLPKDATLKDVVTVIRADEAHHRD

>C. grandiflora (Cagra.1189s0012.1)
GMVGGMLLHCKSLRRFEQSGGWIKALLEEAENERMHLMTFMEVAKPKWYERALVITVQGVFFNAYFLGYLISPKFAHRMVGYLEEEAIHSYTEFLKELDKGNIENVPAPAIAIDYWRLPANATLRDVVMVVRADEAHHRD

>C. grandiflora (Cagra.1189s0011.1)
GMVGGMLVHCKSLRRFEQSGGWIKALLEEAENERMHLMTFMEVAKPNWYERAIVIAVQGVFFNAYFLGYLISPKFAHRMVGYLEEEAIHSYTEFLKELDNGNIENVPAPAIAIDYWRLEADATLRDVVMVVRADEAHHRD

>C. grandiflora (Cagra.3957s0019.1)
GMVGGMLLHLKSLRRFEHSGGWIKALLEEAENERMHLMTFIELSQPKWYERVIVFTVQGVFFNAYFLAYVISPKLAHRITGYLEEEAVNSYTEFLEDIDAGKFENSPAPAIAIDYWRLPKDATLRDVVYVIRADEAHHRD

>C. grandiflora (Cagra.5575s0007.1)
GMVGGMLMHFKSLRRFEQSGGWIKALLEEAENERMHLMTFMEVAKPKWYERALVIAVQGVFFNAYLLGYLISPKFAHRMVGYLEEEAIHSYTEFLKELDNGNIENVPAPAIAIDYWRLEADATLRDVVMVVRADEAHHRD

>B. stricta (Bostr.3359s0124.1)
GMVGGMLLHLKSLRRFEHSGGWIKALLEEAENERMHLMTFTELSQPKWYERAIVFTVQGVFFNAYFLAYVISPKLAHRITGYLEEEAVNSYSEFLKDIDAGKFENSPAPAIAIDYWRLPRDATLRDVVYVIRADEAHHRD

>B. stricta (Bostr.19424s0421.1)
GMVGGMLLHCKSLRRFEQSGGWIKALLEEAENERMHLMTFMEVAKPKWYERALVITVQGVFFNAYFLGYLISPKFAHRMVGYLEEEAIHSYTEFLKELDKGNIENVPAPAIAIDYWRLPADATLRDVVMVVRADEAHHRD

>P. glauca (lcl|PUT-175a-Picea_glauca-1421)
GMVGGMLLHCKSLRKFQHSAGWIKALLEEAENERMHLMTFMEVAQPKWYEKALVFTVQGVFFNAYFLMYIFSPKLAHRIVGYLEEEAIHSYTEFIKELDKGTIPNVPAPAIAIDYWRLPKDSTLRDVVLVIRADEAHHRD

>P. sitchensis (lcl|PUT-183a-Picea_sitchensis-25517)
GMVGGMLLHCKSLRKFQHSAGWIKALLEEAENERMHLMTFMEVTKPNWFERALVFAVQGVFFNTYFLVYIISPKLAHRIVGYLEEEAVYSYTEFLKELDNGNIPNGPAPAIAIDYWRLPKDSTLRDVVMVVRADEAHHRD

>P. banksiana (lcl|PgdbPbanksiana_993)
GMVGGMLLHCKSLRKFQHSGGWIKALLEEAENERMHLMTFMEVAKPRWYERALVFTVQGIFFNAYFLMYILSPKLAHRITGYLEEEAIHSYTEFLKELDKGNIPNVPAPAIAIDYWRLPKDSTLRDVVVVVRADEAHHRD

>P. pinaster (lcl|PgdbPpinaster_2804)
GMVGGMLLHCKSLRKFQHSGGWIKALLEEAENERMHLMTFMEVAKPKWYERALVLTVQGIFFNAYFLMYILSPKLAHRITGYLEEEAIHSYTEFLKELDKGNIPNVPAPAIAIDYWRLPKDSTLRDVVVVVRADEAHHRD

>A. tauschii (EMT10169)

GMVAGAVLHLRSLRRFEQSGGWIRALLEEAENERMHLMTFMEVSQPRWYERALVVAVQGVFFHAYLATYLASPKVAHRMVGYLEEEAVHSYTEFLRDLEAGKIDGVPAPAIAIDYWRLPAGATLKDVVRVVRADEAHHRD

>A. tauschii (EMT02184)

PMVGGVLLHLRSLRRFEHSGGWIRALMEEAENERMHLMTFMDVTQPRWWERALVLAAQGVFFNAYFVGYLISPKFAHRFVGYLEEEAVESYTEYLKDLEAGLIENTPAPAIAIDYWRLPADARLKDVVTAVRADEAHHRD

>A. tauschii (EMT12983)

GMVGGMLLHLRSLRRFEQSGGWIRALLEEAENERMHLMTFMEVAQPRWYERALVIAVQGVFFNAYFFGYLISPKFAHRVVGYLEEEAVHSYTEFLKDLDDGKIDNVPAPAIAIDYWRLPANATLKDVVTVVRADEAHHRD

>T. urartu (TRIUR3_08189-T1)

GMVGGMLLHLRSLRRFEQSGGWIRALLEEAENERMHLMTFMEVANPKWYERALVLAVQGVFFNAYFLGYIVSPKFAHRVVGYLEEEAIHSYTEFLRDLEAGRIENVPAPRIAIDYWRLPADARLKDVVTVVRADEAHHRD

>T. urartu (TRIUR3_19476-T1)

PMVGGVLLHLRSLRRFEHSGGWIRALMEEAENERMHLMTFMEVTQPLWWERALVLATQGVFFNAYFVGYLVSPKFAHRFVGYLEEEAVHSYTEYLKDLEAGLIENTPAPAIAIDYWRLPADARLKDVVTAVRADEAHHRD

>T. urartu (TRIUR3_12374-T1)

PMVGGVLLHLRSLRRFEHSGGWIRALMEEAENERMHLMTFMEVTQPRWWERALVLAAQGVFFNAYFVGYLISPKFAHRFVGYLEEEAVESYTEYLKDLEAGLIENTPAPAIAIDYWRLPADARLKDVVTAVRADEAHHRD

>T. urartu (TRIUR3_10307-T1)

GMVGGMLLHLRSLRRFEQSGGWIRALLEEAENERMHLMTFMEVAQPRWYERALVIAVQGVFFNAYFFGYLISPKFAHRVVGYLEEEAVHSYTEFLKDLDDGKIDNVPAPAIAIDYWRLPANATLKDVVTVVRADEAHHRD

>P. abies (lcl|UCPabies_isotig06527)

GMVGGMLLHCKSLRKFQHSAGWIKALLEEAENERMHLMTFMEVAQPKWYEKALVFTVQGIFFNAYFLMYIFSPKLAHRIVGYLEEEAIHSYTEFIKELDKGTIPNVPAPAIAIDYWRLPKDSTLRDVVLVVRADEAHHRD

>C. mollissima (maker-scaffold13618)

GMVGGMLLHLRSLRKFQQSGGWIKALLEEAENERMHLMTMVELVQPKWYERLLVLTVQGVFFNGFFAVYLLSPKLAHRIVGYLEEEAIHSYTEYLKDIDSGAIENVPAPAIAIDYWKLPKDATLKDVIIVIRADEAHHRD

>C. mollissima (augustus_masked-scaffold22434)

GMVGGMLLHCKSLRRFEHSGGWIKALLEEAENERMHLMTFMEVAKPRWYERALVFTVQGVFFNAYFLGYMISPKFAHRVVGYLEEEAIHSYTEFLKELDKGNIENVPAPAIAIDYWQLPAGSNLRDVVMVVRADEAHHRD

>A. trichopoda (evm_27.model.AmTr_v1.0_scaffold00038.119)
GMVGGLLLHLKSLRKFEHSGGWIKALLEEAENERMHLMTMVEVVKPKWYERLLVLIVQGIFFNAYFILYVLSPKLAHRIVGYLEEEAIHSYTEFLKDLDNGSIPNMAAPAIAIDYWMLPENATLKDVVTVIRADEAHHRD

>A. trichopoda (evm_27.model.AmTr_v1.0_scaffold00048.158)
GMVGGMLLHCKSLRRFEQSGGWIKALLEEAENERMHLMTFMEVAQPKWYERALVITVQGVFFNAYFLMYLISPKLAHRVVGYLEEEAIHSYTEFLKELDNGNIKNVPAPAIALDYWRLPKGSTLRDVVMVVRADEAHHRD

>gi|51860701|gb|AAU11470.1| mitochondrial alternative oxidase 1d [Saccharum officinarum]

GMVGGMLLHLRSLRRFEHSGGWVRALLEEAENERMHLMTFLEVTQPRWWERALVLATQGVFFNAYFVGYLLSPKFAHRVVGYLEEEAVHSYTEYLKDLEAGIIENSPAPAIAIDYWRLPADAKLKDVVTVVRADEAHHRD

>gi|51860695|gb|AAU11467.1| mitochondrial alternative oxidase 1 [Saccharum officinarum]

GMVGGMLLHLRSLRRFEQSGGWIRALLEEAENERMHLMTFMEVAKPRWYERALVITVQGVFFNAYFLGYLLSPKFAHRVVGYLEEEAIHSYTEYLKDVEAGKIENVPAPAIAIDYWRLPANATLKDVVTVVRADEAHHRD

>N. sylvatica (m.23265 g.23265)

GMVGGMLLHLRSLRKFEHSGGWIKALLEEAENERMHLMTMVELVKPKWYERLLVLTVQGVFFNAFFVLYLLSPKLAHRVVGYLEEEAIHSYTEFLKDIDSGAIENVPAPAIAIDYWRLPKDATLKDVITVIRADEAHHRD

>N. sylvatica (m.24086 g.24086)

GMVGGMLLHCKSLRRFEQSGGWIKALLEEAENERMHLMTFIEIGKPKWYERALVFSVQGVFFNAYFLVYLASPKLAHRIVGYLEEEAVNSYTEIIEDLDKGLVENVPAPAIAIDYWRLPPDSTLRDVFVVIRADETHHRD

>Q. rubra (m.24107 g.24107)

GMVGGMLLHCKSLRRFEHSGGWIKALLEEAENERMHLMTFMEVANPRWYERALVFTVQGVFFNAYFLGYLISPKFAHRVVGYLEEEAIHSYTEFLKELDKGNIENVPAPAIAIDYWQLPAGSNLRDVVMVVRADEAHHRD
