# Supplementary material for: Misannotation Awareness: A Tale of Two Gene-Groups
Source: Front Plant Sci. 2016 Jun 16;7:868. doi: 10.3389/fpls.2016.00868 (PMC4909761; doi:10.3389/fpls.2016.00868)
Supplement: Supplementary file 5 [file DataSheet3.ZIP › Read_me.docx]

**On the phylogeny reconstruction approach:**

The protein alignments made available by Costa et al., (2014) as supplementary material were used as the base data for the phylogenetic analysis. To the dataset, other sequences retrieved from the different databases were added including the few existing data on Gymnosperms, giving a total of 369 sequences (full list can be found in Supplementary Table 1). The alignment was performed as in (Costa et al., 2014) (“blosum” substitution matrix with standard gap penalties of pairwise -gap open: 10; gap extension: 0.1- and multiple -gap open: 10; gap extension: 0.2- alignments and 30% of delay divergent cutoff).

**NJ.MTS file**: Phenetic (i.e. based on a single quantitative measure of pairwise similarity) reconstruction of the AOX genes tree - Neigbour-joining with 1000 bootstraps, “number of differences” as the substitution model and “partial deletion” for gaps/missing data treatment in Mega v6.0 (Tamura et al., 2013).

**ML_JTT.MTS file**: For the phylogenetic approach – Maximum Likelihood – The optimal substitution model was selected in MrModeltest 2.2 (Posada and Crandall, 1998) as being the JTT+I+G on the basis of the BIC scores (Bayesian Information Criterion; the lowest the value the better the substitution pattern). Maximum likelihood was also conducted in Mega v6.0, considering the selected model of evolution and 1000 bootstraps.

**BAYES.tre file**: Reconstructed phylogeny of plants at the gene family AOX included in this study (a combination of newly collected sequences and the alignment made available by Costa et al., 2014). The optimal substitution model was selected in MrModeltest 2.2 (Posada and Crandall, 1998) as being the JTT+I+G. The phylogeny corresponds to the majority rule consensus tree of trees sampled in a Bayesian analysis (conducted using MrBayes version 3.0 (Huelsenbeck and Ronquist, 2001; Ronquist and Huelsenbeck, 2003); with default settings and with MCMC -considering 100 000 generations- runs being repeated three times as a safeguard against spurious results; first 1000 trees were discarded as burn-in; stationarity was confirmed by analysis of the log-likelihoods and the consistency between runs).

Costa, J. H., McDonald, A. E., Arnholdt-Schmitt, B., and Fernandes de Melo, D. (2014). A classification scheme for alternative oxidases reveals the taxonomic distribution and evolutionary history of the enzyme in angiosperms. *Mitochondrion*, 1–12. doi:10.1016/j.mito.2014.04.007.

Huelsenbeck, J. P., and Ronquist, F. (2001). MRBAYES: Bayesian inference of phylogenetic trees. *Bioinformatics* 17, 754–755. Available at: http://www.ncbi.nlm.nih.gov/pubmed/11524383.

Posada, D., and Crandall, K. A. (1998). Bioinformatics applications note MODELTEST : testing the model of DNA substitution. 14, 817–818.

Ronquist, F., and Huelsenbeck, J. P. (2003). MrBayes 3: Bayesian phylogenetic inference under mixed models. *Bioinformatics* 19, 1572–1574. doi:10.1093/bioinformatics/btg180.

Tamura, K., Stecher, G., Peterson, D., Filipski, A., and Kumar, S. (2013). MEGA6: Molecular evolutionary genetics analysis version 6.0. *Mol. Biol. Evol.* 30, 2725–2729. doi:10.1093/molbev/mst197.
