# Supplementary material for: Misannotation Awareness: A Tale of Two Gene-Groups
Source: Front Plant Sci. 2016 Jun 16;7:868. doi: 10.3389/fpls.2016.00868 (PMC4909761; doi:10.3389/fpls.2016.00868)
Supplement: Supplementary file 7 [file Image1.PDF]

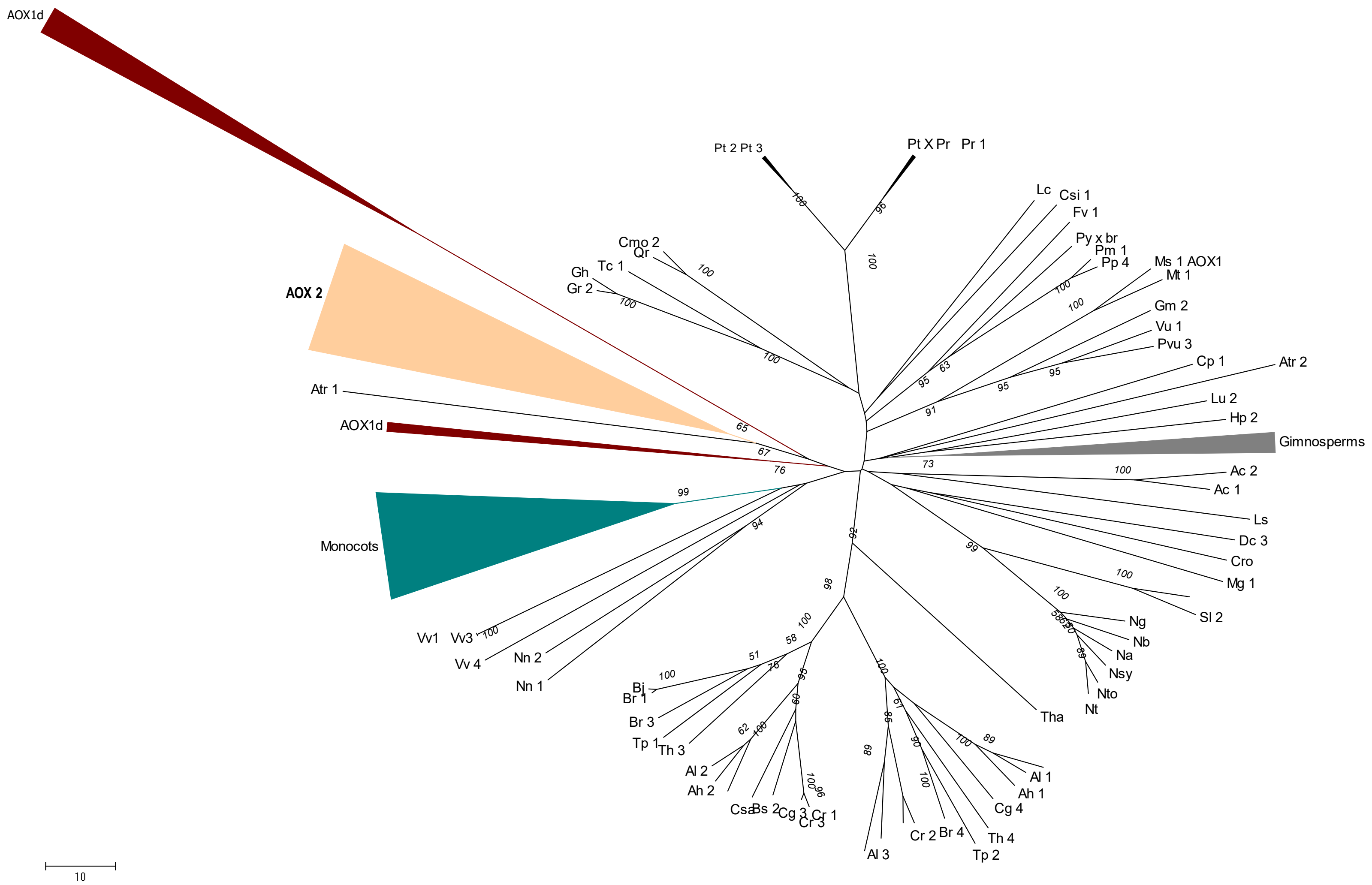

Figure 1: Neighbour-joining tree using the 444 bp amplicon obtained in the *in silico* amplification. A clade comprising AOX2 sequences could be found, separating AOX1 from AOX2 members.
